# Sulforaphane alleviates psoriasis by enhancing antioxidant defense through KEAP1-NRF2 Pathway activation and attenuating inflammatory signaling

**DOI:** 10.1038/s41419-023-06234-9

**Published:** 2023-11-25

**Authors:** Chujun Ma, Chaode Gu, Panpan Lian, Junaid Wazir, Renwei Lu, Binjia Ruan, Lulu Wei, Li Li, Wenyuan Pu, Ziqi Peng, Wentong Wang, Yangyongyi Zong, Zhiqiang Huang, Hongwei Wang, Yan Lu, Zhonglan Su

**Affiliations:** 1 Department of Dermatology, the First Affiliated Hospital of Nanjing Medical University, Nanjing, 210029 PR China; 2 Department of Dermatology, Nanjing Drum Tower Hospital, Affiliated Hospital of Medical School, Nanjing University, Nanjing, PR China; 3 State Key Laboratory of Analytical Chemistry for Life Science & Jiangsu Key Laboratory of Molecular Medicine, Medical School, Nanjing University, Nanjing, 210093 PR China

**Keywords:** Chronic inflammation, Cell biology

## Abstract

Psoriasis is a chronic inflammatory skin disease that affects millions of people worldwide. Sulforaphane (SFN) has been shown to have anti-inflammatory and antioxidant properties. In this study, we investigated the effects of SFN on a mouse model of psoriasis induced by imiquimod (IMQ) and its underlying molecular mechanism. Mice treated with SFN showed significant improvement in psoriatic symptoms, including reduced erythema, scales, and cutaneous thickness. Histopathological analysis and immunohistochemical staining revealed decreased expression of K16, K17, and Ki67 in SFN-treated mice, indicating reduced abnormal differentiation of keratinocytes and cutaneous inflammation. SFN treatment also reduced the activation of STAT3 and NF-κB pathways and downregulated pro-inflammatory cytokines IL-1β, IL-6, and CCL2. In vitro experiments using HaCaT cells demonstrated that SFN inhibited IL-22 and TNF-α-induced activation of inflammatory pathways and keratinocyte proliferation. Network pharmacology analysis suggested that the KEAP1-NRF2 pathway might be involved in the protective effects of SFN on psoriasis. We observed reduced NRF2 expression in human psoriatic lesions, and subsequent experiments showed that SFN activated KEAP1-NRF2 pathway in vivo and in vitro. Importantly, NRF2-deficient mice exhibited aggravated psoriasis-like symptoms and reduced response to SFN treatment. Our findings indicate that SFN ameliorates psoriasis symptoms and inflammation through the KEAP1-NRF2 pathway, suggesting a potential therapeutic role for SFN in the treatment of psoriasis.

## Introduction

Psoriasis is a common long-lasting cutaneous inflammatory disease that affects 3–4% of the global population. Its clinical symptoms are typically red patches with white scales on the top, which appear on the elbows, knees, scalp, and back [1]. It is widely accepted that multiple factors, including genetics and environment, contribute to the pathological development of psoriasis, but its etiology is complex and poorly understood [2].

Oxidative stress is a risk factor for this dermatosis [3]. Oxidative stress refers to a state where there is an elevated production of reactive oxygen species (ROS) or reactive nitrogen species (RNS), coupled with a reduced concentration or diminished activity of antioxidants responsible for neutralizing these reactive species. ROS can damage DNA, proteins, and lipids and activate a variety of inflammatory pathways, all of which contribute to psoriatic inflammation [4, 5]. Patients with psoriasis have increased ROS production and decreased antioxidant capacity [6]. In addition, the severity and duration of the disease are correlated with the level of oxidative stress in the body [7]. The production of ROS and free radicals exacerbates inflammation and hyperplasia of keratinocyte cells [8]. In turn, keratinocytes, activated neutrophils, and lymphocytes in the skin lesions of psoriasis result in the generation of abundant amounts of ROS and free radicals [9]. Therefore, supplementation with exogenous antioxidants represents a useful adjunct option in the treatment of psoriasis.

Sulforaphane (SFN) is a natural isothiocyanate group of organosulfur compounds presents in cruciferous vegetables. In recent years, it has been extensively studied for its potent therapeutic effects on diseases and low cytotoxicity. The antioxidant properties of SFN have been demonstrated to inhibit cell proliferation, activate caspases, induce apoptosis, and stop the cell cycle [10–12]. In a variety of cancers and chronic inflammatory diseases, SFN has been proven to have promising therapeutic effects [13, 14]. Mechanistic studies indicated that SFN regulated the expression of various tumor suppressor genes or inflammatory genes by targeting the HDAC, DNMT, and NF-κB signaling pathways. Additionally, NRF2 and several microRNAs have been shown to be regulated by SFN [13, 15]. However, the functional role and the involved mechanism of SFN in psoriasis are not known and still need to be defined.

In the present study, we aimed to investigate the modulatory effects of SFN in imiquimod (IMQ)-induced psoriatic mice, as well as the underlying molecular mechanisms. We found that decreased NRF2 expression and activation resulted in increased oxidative stress in psoriatic lesional skin. The application of SFN significantly protected cutaneous inflammation in an NRF2-dependent manner. Our data provide crucial experimental evidence that SFN could be used as a therapeutic compound for the treatment of psoriasis.

## Methods

### Skin specimens from psoriasis patients and healthy donors

Skin biopsy specimens were procured from five individuals recently diagnosed with psoriasis and five healthy participants undergoing cosmetic plastic surgery procedures. Diagnoses were made through a combination of clinical assessments and histological analyses, with none of the patients having received any systemic or topical treatments for a minimum of 2 weeks prior to the biopsy. The research protocol received ethical clearance from the Institutional Review Board of the First Affiliated Hospital of Nanjing Medical University. All those contributing skin samples provided informed written consent, and both the patients and volunteers expressed agreement for the publication of their photographs.

### IMQ-induced psoriatic mouse model

Six-week-old female BALB/c mice or C57BL/6 mice, along with NRF2 knockout mice (acquired from the Model Animal Research Center of Nanjing University), were randomly grouped and maintained in an SPF environment, subjected to a 12-hour light/dark cycle, and provided with unrestricted access to food and water.

The Nanjing University Institutional Animal Ethics Committee granted approval for all animal experiments. The IMQ-induced psoriasis mouse model was developed according to previously established methods. In brief, the mice were administered a daily topical application of 62.5 mg of 5% imiquimod cream (Mingxinlidi, Sichuan, China) on their shaved backs for seven consecutive days. The same amount of vaseline cream was used on control mice and the SFN alone group. SFN (MedChemExpress, NJ, USA) was intraperitoneally injected at 5 mg/kg once a day for 7 consecutive days. The same dose of normal saline was used on control mice and IMQ-induced psoriatic mice. The severity of cutaneous inflammation was scored 24 h after the last drug administration using the Psoriasis Area and Severity Index (PASI), scored by two scorers who were blind to the experimental groups: 0, none; 1, 2, moderate; 3, severe; 4, very severe. The mice were euthanized by cervical dislocation and the spleen was weighed to calculate the spleen index: spleen index = spleen weight (g)/mouse weight (g).

### Western blot analysis

To quantify the protein samples, tissue homogenates and cells were homogenized and quantified using a BCA kit (Beyotime, Shanghai, China). The protein samples were separated using 10% SDS-polyacrylamide gels and transferred to PVDF membranes in equal amounts. After incubating with 5% bovine serum albumin for 1 h, the membranes were incubated with primary antibodies overnight at 4 °C. Antibodies used were: anti-keratin 16 (sc-53255, Santa Cruz); anti-keratin 17 (ab53707, Abcam, Cambridge, UK), anti-NRF2 (#12721, Cell Signaling Technologies, MA, USA), anti-phospho-NRF2 (AF1609, Beyotime, Shanghai, China), anti-IL-1β (#12703, Cell Signaling Technologies, MA, USA), anti-NQO1 (AF7614, Beyotime, Shanghai, China), anti-phospho-STAT3 (Tyr705) (#9145, Cell Signaling Technology, MA, USA), anti-STAT3 (#12640, Cell Signaling Technology, MA, USA), anti-phospho-NF-κB p65 (#3033, Cell Signaling Technology, MA, USA), anti-NF-κB p65 (#8242, Cell Signaling Technology, MA, USA), anti-phospho-IκBα (#2859, Cell Signaling Technology, MA, USA), anti-IκBα (#4812, Cell Signaling Technology, MA, USA) and GAPDH (BS60630, Bioworld Technology Inc., MN, USA). Blots were incubated with HRP-conjugated secondary antibodies (BS13278, Bioworld Technology Inc., MN, USA) for 1 h, and protein expression was detected with ECL (WBULS0500, Millipore, MA, USA) by digital imaging systems (SYNGENE, Cambridge, UK). ImageJ software was used to analyze the density of the western blot results.

### Histopathological examination and immunofluorescence staining

The dorsal skin was preserved in 10% buffered formalin for a duration of 24 h before being embedded in paraffin wax. Paraffin-embedded samples were sectioned into 3-micrometer-thick slices and mounted onto glass slides. These slides were then subjected to hematoxylin and eosin (H&E) staining and immunohistochemical analysis, adhering to the guidelines outlined in the AFIP Laboratory Methods in Histotechnology manual. The streptavidin-peroxidase technique was employed for immunostaining. Primary antibodies used included anti-keratin 16 (sc-53255, Santa Cruz), anti-Ki67 (ab16667, Abcam, Cambridge, UK), anti-keratin 17 (ab53707, Abcam, Cambridge, UK), and anti-NQO1 (AF7614, Beyotime, Shanghai, China).

### Real-Time Quantitative Reverse Transcription PCR (qRT‒PCR) Analysis

Total RNA from skin tissues was isolated using TRIzol reagent (Sigma, Milwaukee, WI, USA), and 1 μg of total RNA was reverse-transcribed into cDNA. All qRT‒PCR assays were performed on the QuantStudio 5 Real-Time PCR system (Applied Biosystems, CA, USA) with Power-Up SYBR Master Mix (Applied Biosystems, CA, USA). The relative expression of the target genes was normalized to that of *Actb* or *GAPDH* and calculated with the 2ΔΔCt method. The primer sequences of the target and control genes were as follows: *Il6* forward: 5′-ACA AAG CCA GAG TCC TTC AGA G-3′ and reverse, 5′-GGT CCT TAG CCA CTC CTT CTG-3′; *Il1b* forward: 5′-TCC AGG ATG AGG ACA TGA GCA-3′ and reverse, 5′-GAA CGT CAC ACA CCA GCA GGT-3′; *Ccl2* forward: 5′-TTA AAA ACC TGG ATC GGA ACC AA-3′ and reverse, 5′-GCA TTA GCT TCA GAT TTA CGG GT-3′; *Il17a* forward: 5′-TGA CCC CTA AGA AAC CC CCA-3′ and reverse, 5′-TCA TTG TGG AGG GCA GAC AA-3′; *Il23a* forward: 5′-ATG CTG GAT TGC AGA GCA GTA-3′ and reverse, 5′-ACG GGG CAC ATT ATT TTT AGT CT-3′; *Actb* forward: 5′-CTA AGG CCA ACC GTG AAA AG-3′ and reverse, 5′-ACC AGA GGC ATA CAG GGA CA-3′; *NFE2L2* forward: 5′-TCA GCG ACG GAA AGA GTA TGA-3′ and reverse, 5′-CCA CTG GTT TCT GAC TGG ATG T-3′; *GAPDH* forward: 5′-GTC TCC TCT GAC TTC AAC AGC G-3′ and reverse, 5′-ACC ACC CTG TTG CTG TAG CCA A-3′.

### Cell culture and treatment

Immortalized human keratinocyte (HaCaT) cells (NE Fusenig, Heidelberg, Germany) were cultured in adipocyte medium (AM, high-glucose DMEM with 10% FBS and 1% P/S) at 37 °C in a humidified incubator with 5% CO_2_. HaCaT cells were treated with IL-22 (25 mmol/L; 13059-HNAE, Sino Biological, China) or TNF-α (25 mmol/L; 10602-H01H, Sino Biological, China) for 30 min or 24 h to establish a psoriasis-like keratinocyte model.

### Lentiviral-based transfection into HaCaT cells

For suppression of NRF2 in HaCaT cells, lentiviral particles expressing shRNAs against human NRF2 were used to downregulate NRF2 mRNA. The knockdown system was performed according to the Addgene standard protocol [16]. Transduced cells were selected with 2 µg/ml puromycin (Sigma-Aldrich, TR-1003-G).The DNA sequences of these shRNA genes were as follows: sh-NRF2-a forward: 5′- CCG GCT TGC ATT AAT TCG GGA TAT ACT CGA GTA TAT CCC GAA TTA ATG CAA GTT TTT G-3′ and reverse, 5′- AAT TCA AAA ACT TGC ATT AAT TCG GGA TAT ACT CGA GTA TAT CCC GAA TTA ATG CAA G-3′, sh-NRF2-b forward: 5′-CCG GAG AGC AAG ATT TAG ATC ATT TCT CGA GAA ATG ATC TAA ATC TTG CTC TTT TTT G-3′ and reverse, 5′- AAT TCA AAA AAG AGC AAG ATT TAG ATC ATT TCT CGA GAA ATG ATC TAA ATC TTG CTC T-3′, sh-C forward: 5′- CCG GGC AAG CTG ACC CTG AAG TTC ACT CGA GTG AAC TTC AGG GTC AGC TTG CTT TTT G-3′ and reverse, 5′- AAT TCA AAA AGC AAG CTG ACC CTG AAG TTC ACT CGA GTG AAC TTC AGG GTC AGC TTG C-3′.

### Measurements of intracellular ROS

After treatment with IL-22 (25 ng/ml) and/or SFN (20 nM), HaCaT cells were harvested and stained with 2 µM dihydroethidium (DHE, Beyotime Institute of Biotechnology) for 30 min at 37 °C in the dark. The fluorescence intensity was assayed by an FV10i laser scanning confocal microscope (Olympus, Tokyo, Japan).

### Target analysis of sulforaphane in psoriasis

To study the molecular mechanism by which SFN alleviates psoriasis, network pharmacology was performed using the SuperPred (https://prediction.charite.de/) and OMIM (https://www.omim.org/) databases to predict the target molecules of SFN. The target molecules were converted to genes and intersected with psoriasis-related genes from GeneCards (https://www.genecards.org/) and OMIM. The PPI network of the target genes was created using STRING (https://cn.string-db.org/), and the pathways were assessed via Reactome analysis for enrichment. The expression levels of NRF2 were evaluated using the Human Protein Atlas portal website (http://www.proteinatlas.org/) and the Gene Expression Omnibus (GEO), GSE119087, database.

### Statistical analyses

Statistical analyses were conducted using GraphPad Prism software (version 9.0.0). Dual comparisons were unpaired Student’s *t*-tests, and multigroup comparisons were made with one-way analysis of variance (ANOVA). The results of three independent experiments are presented as the mean ± standard deviation (SD), and *p* < 0.05 was considered statistically significant.

## Results

### SFN ameliorates psoriatic symptoms in IMQ-induced psoriatic mouse model

**Fig. 1:**
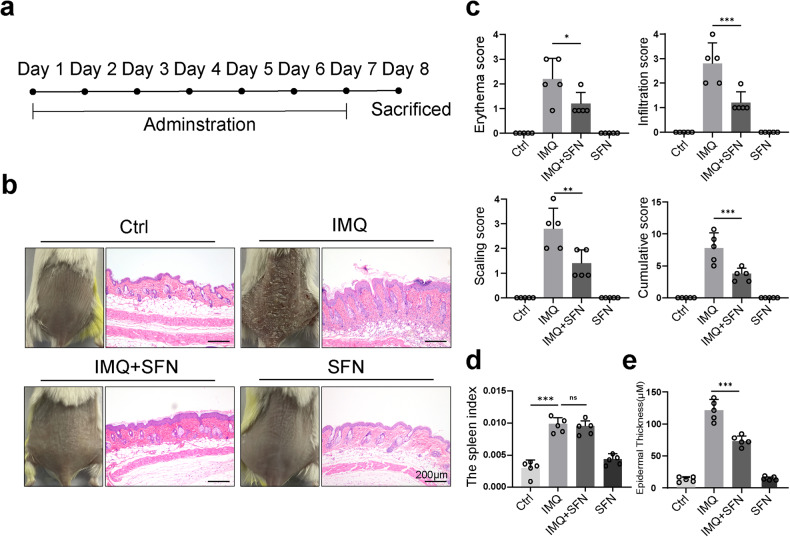
Sulforaphane (SFN) ameliorates psoriatic symptoms in IMQ-induced psoriatic mouse model. **a** Mice were treated with IMQ and sulforaphane daily for 7 consecutive days and sacrificed on day 8. *n* = 5. **b** The macroscopic appearance and H&E staining (magnification × 100) of the skin tissue. **c** The PASI scores of the skin tissue. **d** Spleen weight index, which was measured by taking spleen weight to body weight. **e** The average epidermal thickness (μM), derived from 18–20 random measurements. Statistical significance was determined by one-way ANOVA analysis. *p < 0.05, **p < 0.01 and ***p < 0.001.

To investigate the functional role of SFN in psoriasis, a mouse model of IMQ-induced psoriasis treated with systemic SFN was used (Fig. 1a). Mice that received 5% IMQ cream applied to shaved dorsal skin daily for seven consecutive days developed a distinct psoriasis-like appearance (Fig. 1b, left panel). Psoriatic mice treated with SFN, however, showed a significant decrease in IMQ-induced psoriatic traits, such as erythema, scales, and cutaneous thickness. Furthermore, mice treated with SFN had fewer psoriasiform lesions (Fig. 1b, left panel). The PASI scores calculated and plotted on day 8 are shown in Fig. 1c. Moreover, SFN treatment significantly reduced the overall PASI score compared with the IMQ group, indicating the protective effects of SFN on psoriasis.

To further evaluate the role of SFN in the IMQ-induced psoriasis mouse model, histopathological analysis of mouse dorsal skin was performed by H&E staining (Fig. 1b, right panel). Mice in the IMQ group developed typical psoriasis-like changes, such as Munro microabscesses in the corneum, hyperkeratosis and parakeratosis in the epidermis, spinous layer thickening, regularly elongated rete ridges, and lymphocytes infiltration in the dermis (Fig. 1b, right panel). In contrast, the SFN-treated group showed remarkable improvement in epidermal thickness (Fig. 1e) and infiltrating cells (Fig. 1b, right panel), although no significant difference was observed in spleen size (Fig. 1d).

### Psoriatic mice treated with SFN exhibit decreased keratinocyte differentiation and reduced cutaneous inflammation

**Fig. 2:**
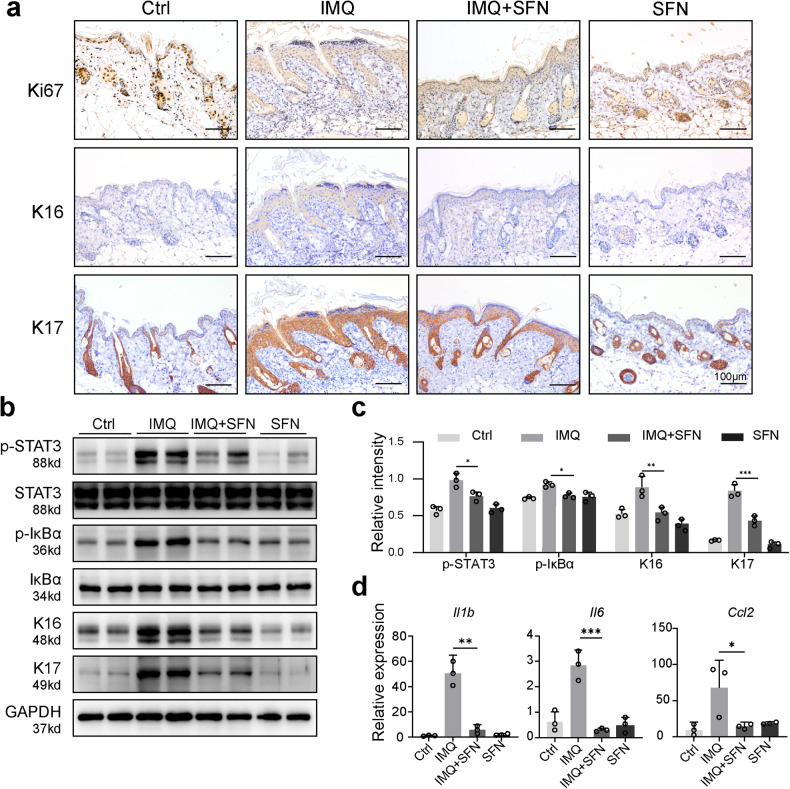
Psoriatic mice treated with SFN exhibit decreased keratinocyte differentiation and reduced cutaneous inflammation. **a** The contents of Ki67, keratin 16 (K16) and keratin 17 (K17) in the dorsal epidermis were measured using immunohistochemical staining (magnification × 200). **b** -STAT3, STAT3, p-IκBα, κBα, K16 and K17 expression was detected using Western blotting. **c** The relative expression levels of p-STAT3, p-IκB, K16 and K17 were analyzed by normalization to GAPDH. **d** Quantitative real-time polymerase chain reaction (qRT–PCR) was performed to evaluate the effects of SFN on the mRNA expression of *Il1b*, *Il6* and *Ccl2* in psoriatic mouse skin. *n* = 3. Statistical significance was determined by one-way ANOVA analysis. *p < 0.05, **p < 0.01 and ***p < 0.001.

Keratin 16 (K16) and keratin 17 (K17) are established markers of excessive keratinocyte hyperproliferation in psoriasis and are known to promote T-cell activation and psoriasis development [17, 18]. Ki67 serves as a cell proliferation marker for keratinocytes [19]. We investigated the expression of K16, K17, and Ki67 in mouse skin tissues using immunohistochemical staining. Our findings demonstrated that the SFN-treated group exhibited significantly lower expression of K16, K17, and Ki67 compared to the IMQ group (Fig. 2a). Moreover, SFN treatment notably decreased the elevated protein expression levels of K16 and K17 (Fig. 2b, c).

Persistent STAT3 activation, a recognized potential target in psoriasis, is crucial in psoriasis pathogenesis. Furthermore, NF-κB is a key transcription factor in immune response, with numerous downstream genes in the NF-κB pathway contributing to psoriasis’ pathological development [20, 21]. To examine the impact of SFN on inflammatory pathways in psoriatic mouse skin, we measured the expression levels of p-STAT3 and p-IκBα. The IMQ group displayed significantly increased expression levels of p-STAT3 and p-IκBα. In contrast, SFN treatment considerably diminished the expression levels of p-STAT3 and p-IκBα in psoriatic mouse skin (Fig. 2b, c).

To further verify whether SFN could modulate the expression of inflammatory factors and chemokines during psoriasis development, we evaluated the mRNA levels of *Il1b*, *Il6*, and *Ccl2* using qRT‒PCR. IL-1β, which is secreted by macrophages and has a vital role in psoriasis immunity [22], and IL-6, which activates the JAK-STAT pathway and exhibits elevated levels in psoriasis patients’ lesions [23], were both significantly increased in psoriasis-induced mice. However, SFN treatment substantially reduced *Il1b* and *Il6* mRNA expression levels (Fig. 2d). Additionally, CCL2, a neutrophil chemokine implicated in angiogenesis and the pathological changes of psoriatic lesions [24], was markedly elevated in psoriasis-induced mice. SFN treatment effectively decreased the mRNA expression of *Ccl2* compared to psoriasis-induced mice (Fig. 2d). These findings highlight the efficacy of SFN treatment in attenuating keratinocyte hyperproliferation and cutaneous inflammation in psoriatic mice by regulating the expression of crucial inflammatory markers and chemokines, indicating its potential therapeutic value in managing psoriasis.

### SFN mitigates TNF-a and IL-22-induced activation of inflammatory pathways and proliferation in HaCaT cells

**Fig. 3:**
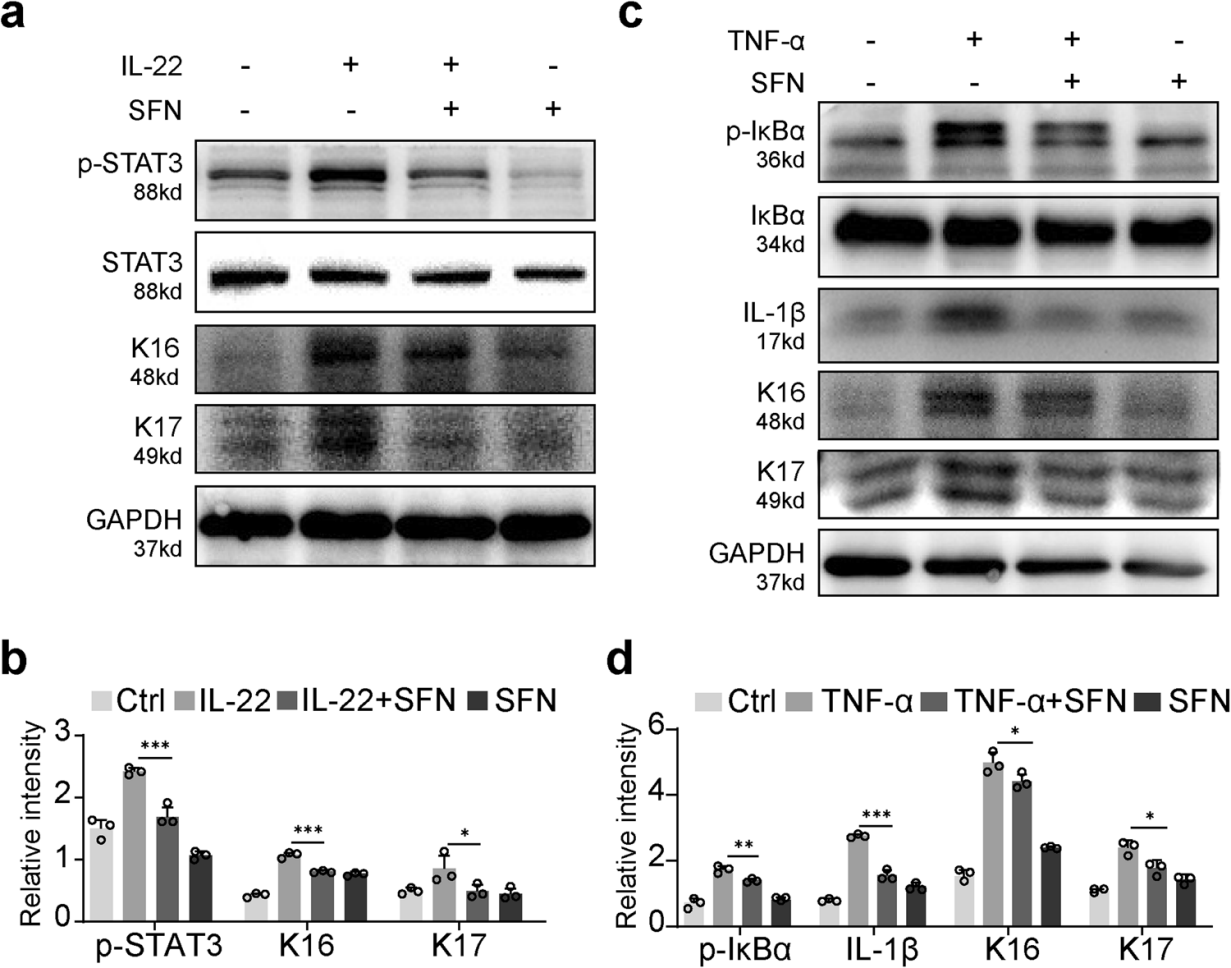
SFN mitigates TNF-a and IL-22-induced activation of inflammatory pathways and proliferation in HaCaT cells. **a** The expression of p-STAT3, STAT3, K16, and K17 in response to SFN and IL-22 (25 ng/ml) treatment was determined by western blotting. **b** The relative expression levels were analyzed by normalization to GAPDH. **c** Western blot analysis of the protein expression of p-IκBα, IκBα, IL-1β, K16 and K17. **d** The relative protein levels were assessed by ImageJ software. Statistical significance was determined by one-way ANOVA analysis. *p < 0.05, **p < 0.01 and ***p < 0.001.

IL-22 is known to bind to the IL-22 receptor on keratinocytes, induce STAT3 phosphorylation, and promote keratinocyte proliferation [25, 26]. Considering the significant role of IL-22 in psoriasis pathogenesis, we added IL-22 (25 mM) to HaCaT cells treated with or without SFN. We then detected the protein expression levels of STAT3, p-STAT3, K16, and K17 using western blot analysis. Our results revealed that SFN treatment considerably inhibited the phosphorylation of STAT3 and the expression of K16 and K17 (Fig. 3a, b).

TNF-α-induced NF-κB pathway activation plays a part in psoriasis, and TNF-α monoclonal antibodies have been employed in clinical settings for psoriasis treatment [27]. To investigate the impact of SFN, HaCaT cells were treated with TNF-α (25 mM). In line with previous findings, SFN impeded TNF-α-induced phosphorylation of IκB and significantly decreased IL-1β expression. Additionally, TNF-α stimulated the expression of K16 and K17, whereas SFN suppressed their expression (Fig. 3c, d). These results suggest that SFN exerts an inhibitory effect on both the STAT3 and NF-κB signaling pathways.

### SFN alleviates psoriasis via the KEAP1-NFE2L2 pathway

**Fig. 4:**
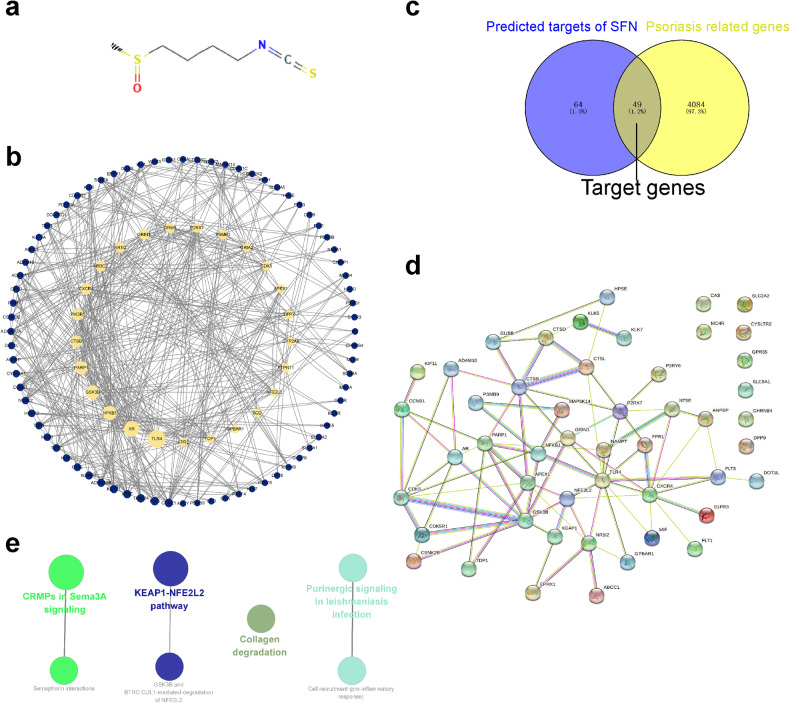
Target analysis of SFN in psoriasis. **a** Chemical structure of SFN. **b** PPI network of SFN targets predicted by SuperPred(https://prediction.charite.de/) and OMIM (https://www.omim.org/). The yellow nodes represent the large hub nodes, and the blue nodes represent the other nodes. The node size is proportional to the target degree in the network. **c** Target genes were retrieved by taking the intersection of targets of SFN and genes related to psoriasis, and the PPI network of the target genes (**d**) was constructed by the STRING database. **e** Interrelation analysis of pathways via assessment of Reactome processes in ClueGO.

Given the observed therapeutic effects of SFN, we sought to investigate the potential molecular mechanisms responsible for the therapeutic effect. Network pharmacology was employed to investigate the molecular mechanism of SFN in treating psoriasis. Using the SuperPred and OMIM databases, 113 target molecules were identified (Fig. 4a) and converted to corresponding genes, which were then cross-referenced with psoriasis-related genes from GeneCards and OMIM. Forty-nine target genes were found (Fig. 4b). The PPI network was constructed with STRING (Fig. 4c) and Reactome pathway analysis in ClueGO was utilized to evaluate the target genes. Four significant pathways were identified: CRMPs in Sema3A signaling, KEAP1-NFE2L2 pathway, Purinergic signaling in leishmaniasis infection, and Collagen degradation (Fig. 4d). The KEAP1-NFE2L2 pathway, with the most enriched genes, was selected for further study.

**Fig. 5:**
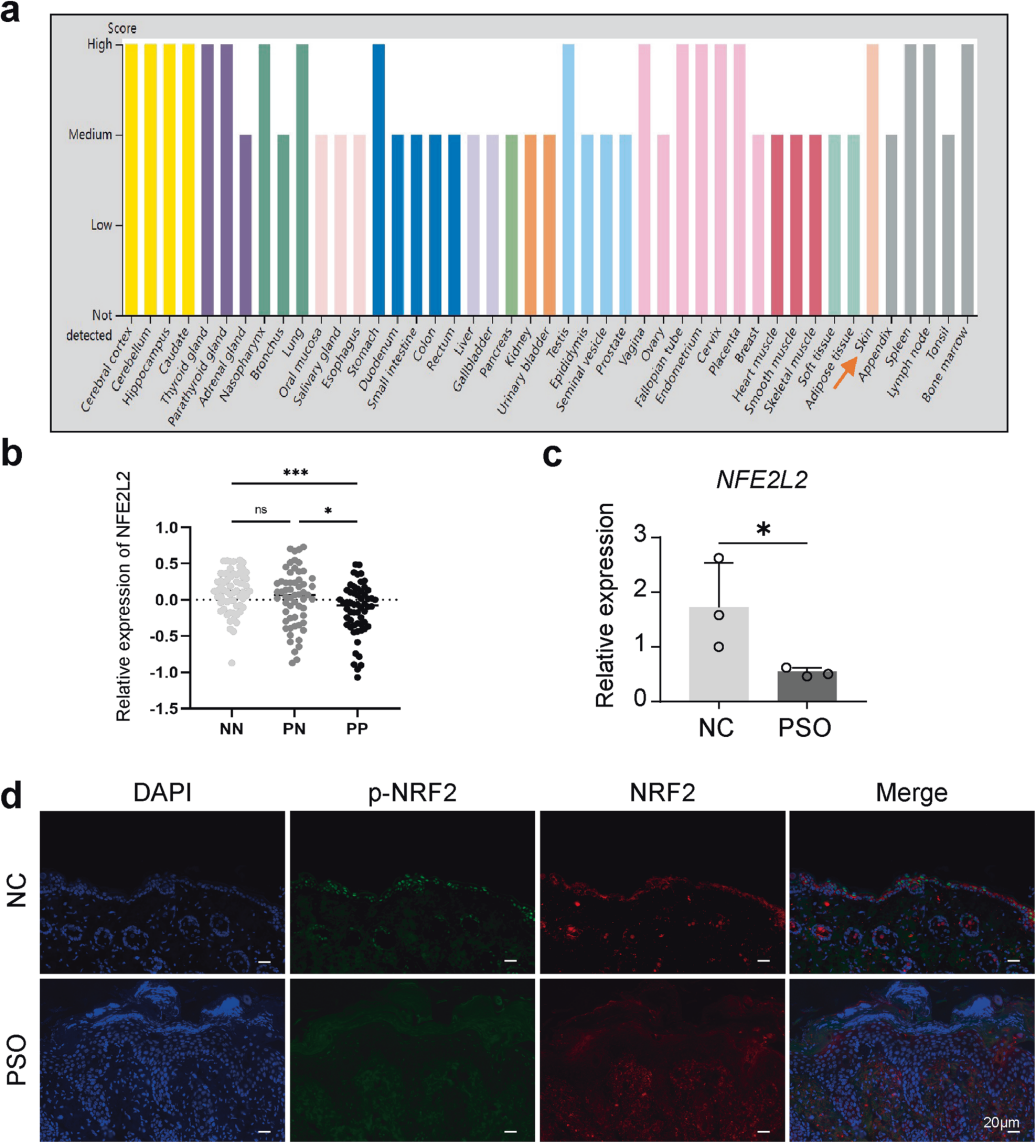
NRF2 expression is decreased in psoriatic skin compared to normal skin. **a** NRF2 protein expression was elevated in skin tissue (arrows) from the Human Protein Atlas website. The orange arrow indicates the levels of NRF2 in skin tissue. **b** *NFE2L2* mRNA levels of normal skin (NN), normal skin in patients with psoriasis (PN) and psoriatic skin in patients with psoriasis (PP) expressed in skin tissue from GEO datasets (GSE119087). **c** RT–PCR was performed to evaluate the mRNA level of *NFE2L2* in psoriatic human skin (PSO) and skin from healthy tissue (NC). **d** The protein level of p-NRF2 and NRF2 in PSO and NC was determined by immunofluorescence analysis. *p < 0.05, **p < 0.01 and ***p < 0.001.

**Fig. 6:**
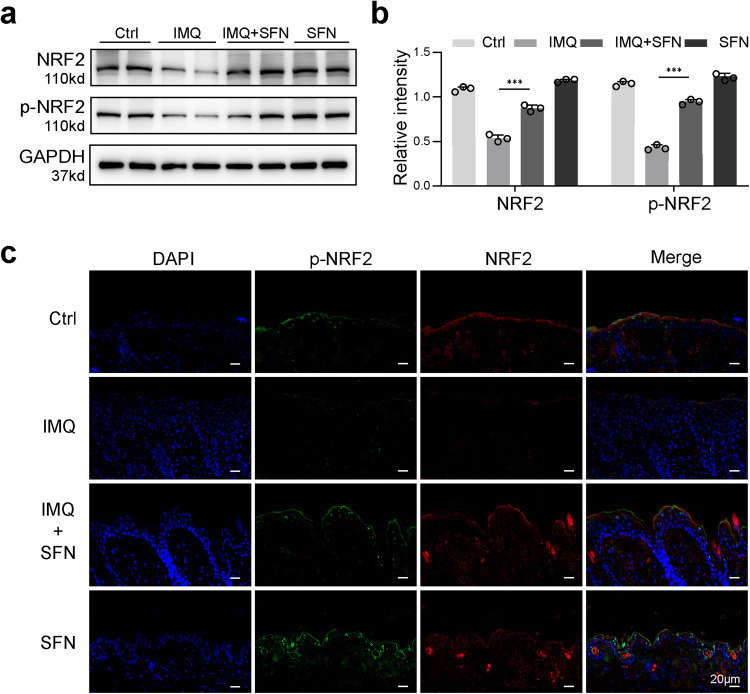
SFN increases the expression of NRF2 and p-NRF2 in IMQ-induced psoriatic mouse model. **a** Protein expression of NRF2 and p-NRF2 evaluated using Western blot. **b** The relative expression levels were analyzed by normalization to GAPDH. ***p < 0.001. **c** Immunofluorescence staining for NRF2 and p-NRF2 on skin sections of flaky mice was performed.

**Fig. 7:**
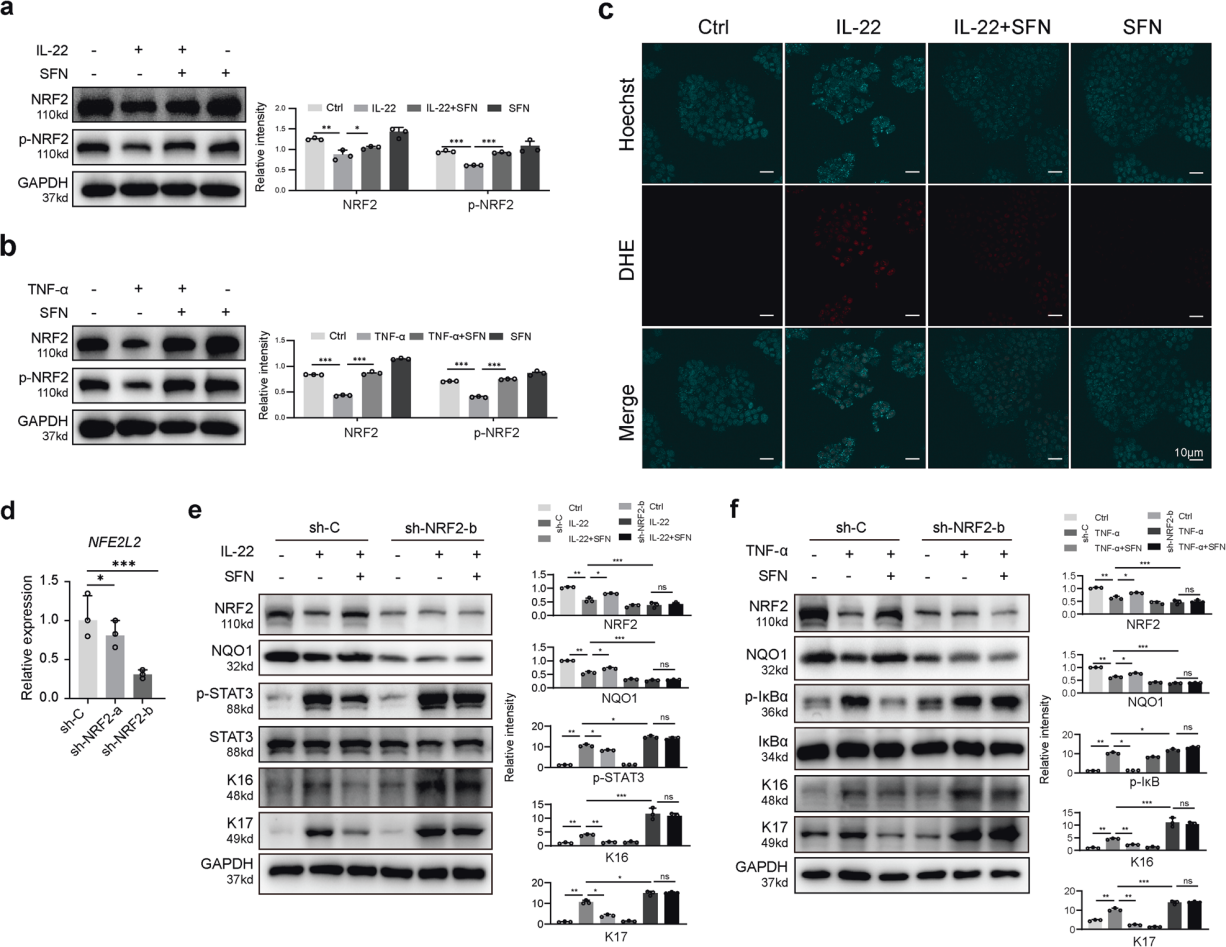
NRF2 pathway was involved in the cytoprotection of SFN against IL-22 and TNF-α. **a** The expression of NRF2 and p-NRF2 in response to SFN and IL-22 treatment was determined by western blotting. **b** The expression of NRF2 and p-NRF2 in response to SFN and TNF-α treatment was determined by western blotting. **c** HaCaT cell superoxide measured by staining with DHE (magnification × 400). **d** HaCaT cells were transduced with lentivirus carrying shRNA against a scramble sequence (sh-C) or against NRF2(sh-NRF2-a and sh-NRF2-b). Expression levels of *NFE2L2* were determined by qRT–PCR and normalized to Actb levels. **e** The Western Blot results of SFN and IL-22 treatment in the NRF2 knockdown group (sh-NRF2-b) and the control group (sh-C). **f** The Western Blot results of SFN and TNF-α treatment in the NRF2 knockdown group (sh-NRF2-b) and the control group (sh-C). *p < 0.05, **p < 0.01 and ***p < 0.001.

NRF2 is a bZIP protein involved in regulating oxidative stress, inflammation, autophagy, and immune response. NRF2 is anchored in the cytoplasm under normal conditions but enters the nucleus and activates antioxidant genes during oxidative stress [28, 29]. We used the Human Protein Atlas and GEO database to observe NRF2 expression in various organ tissues, including skin, and found decreased *NFE2L2* expression in psoriatic lesions (Fig. 5a, b). qRT‒PCR and immunofluorescence confirmed reduced *NFE2L2* RNA and protein expression in psoriatic lesions compared to normal skin (Fig. 5c, d). In vivo treatment with SFN resulted in higher NRF2 and p-NRF2 expression in SFN-treated mice compared to IMQ-induced psoriatic mice (Fig. 6a–c). In vitro, SFN pretreatment restored NRF2 and p-NRF2 expression in IL-22-treated and TNF-α-treated HaCaT cells (Fig. 7a, b). Moreover, SFN treatment reduced IL-22-induced superoxide production (Fig. 7c). These findings suggest that NRF2 has a protective role in psoriasis and that SFN enhances NRF2 and p-NRF2 expression. We established a stable NRF2 knockdown HaCaT cell line using lentivirus-mediated techniques to delve deeper into NRF2’s role in skin inflammation. Lentivirus containing shRNA targeting either a non-specific scramble sequence (sh-C) or specifically targeting NRF2 (sh-NRF2-a and sh-NRF2-b) was used to transduce HaCaT cells. Subsequently, we assessed *NFE2L2* expression using qRT‒PCR (Fig. 7d) and proceeded with experiments utilizing cells infected with sh-NRF2-b. In vitro experiments revealed that, under non-inflammatory conditions, NRF2 knockdown did not induce activation of the STAT3 or NF-κB pathways, nor did it affect the expression of K16 and K17. However, upon the addition of IL-22 (Fig. 7e) or TNF-α (Fig. 7f), cells infected with sh-NRF2-b exhibited significantly enhanced activation of the STAT3 and NF-κB pathways, accompanied by a marked increase in K16 and K17 expression. Notably, this trend remained unaltered even after the introduction of SFN.

### NRF2 depletion exacerbates IMQ-induced psoriasis-like symptoms and negates the therapeutic effect of SFN

**Fig. 8:**
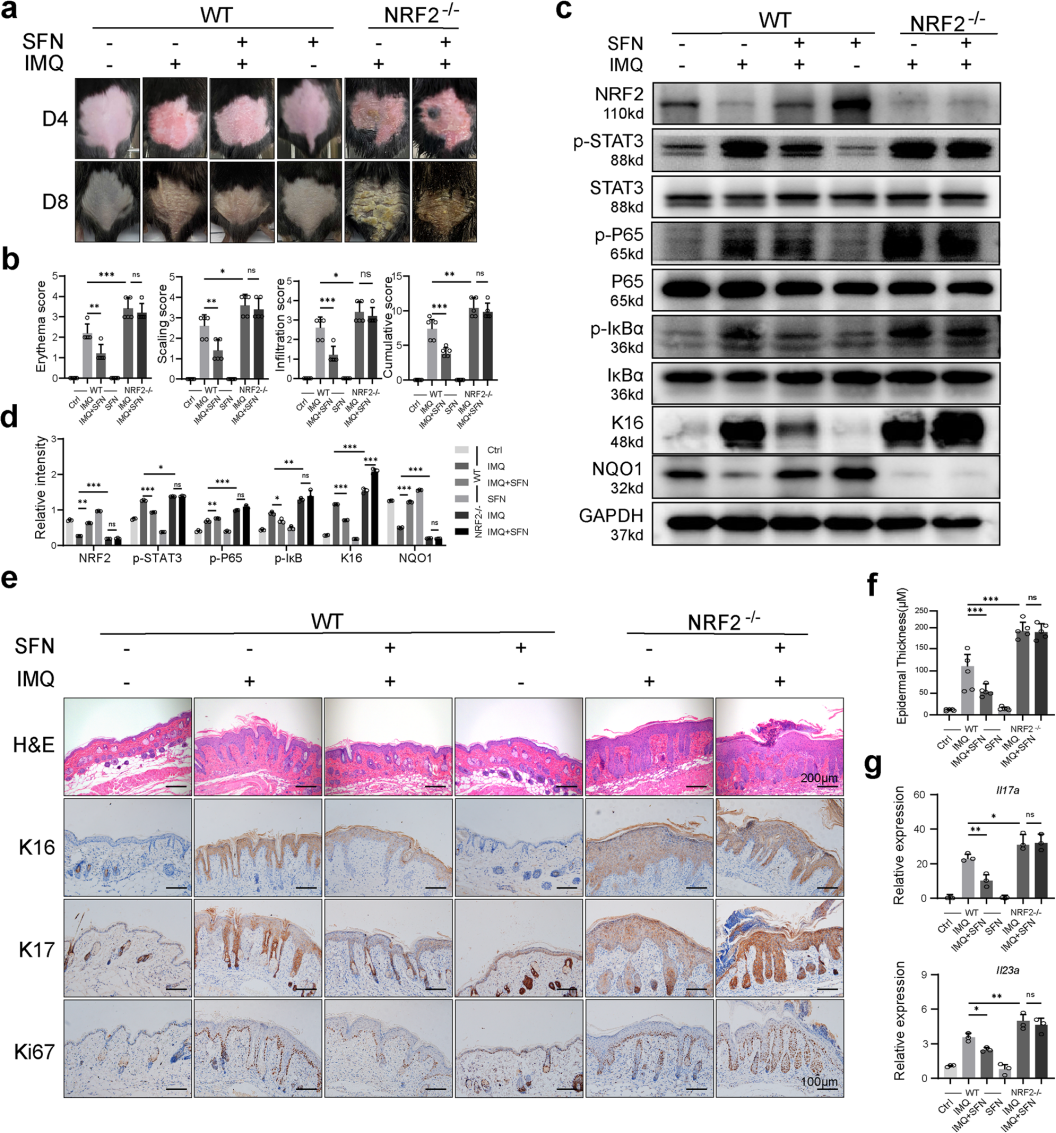
NRF2 depletion exacerbates IMQ-induced psoriasis-like symptoms and negates the therapeutic effect of SFN. **a** Wild-type (WT) and NRF2^−/−^ mice were topically treated with IMQ for seven consecutive days and sacrificed on day 8. Images were taken on day 4 and day 8. *n* = 5. **b** The PASI scores of the skin tissue. **c** p-STAT3, STAT3, p-P65, P65, p-IκBα, IκB, K16 and NQO1 expression was detected using Western blotting. **d** The relative expression levels of protein were analyzed by normalization to GAPDH. **e** H&E staining (magnification × 100) and immunohistochemical staining of Ki67, K17 and K16 (magnification × 200) in skin sections of mice were performed. **f** The average epidermal thickness (μM), derived from 18–20 random measurements. **g** qRT–PCR was performed to evaluate the mRNA expression of *Il17a* and *Il23a* in mouse skin. *n* = 3. *p < 0.05, **p < 0.01 and ***p < 0.001.

**Fig. 9:**
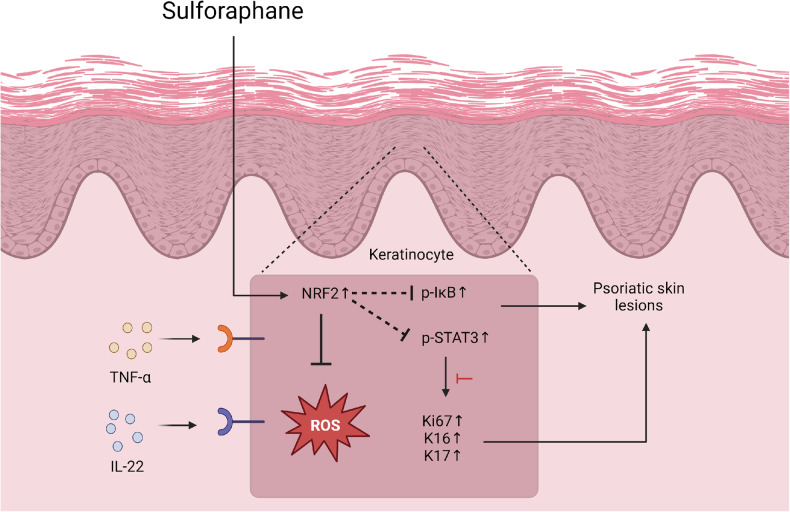
The mechanism driving the therapeutic impact of SFN in combating psoriasis. Diminished expression of NRF2 plays a role in oxidative stress injury in psoriasis. By activating the NRF2 signaling pathway, SFN mitigates oxidative stress injury and reduces the inflammatory response associated with psoriasis.

To further investigate the mechanism of SFN, we utilized both wild-type and NRF2-deficient mice for modeling and treatment. The NRF2-deficient mice did not spontaneously develop skin lesions (Fig. S1). On days 4 and 8, NRF2-deficient mice displayed more severe lesions that did not show significant improvement following SFN treatment (Fig. 8a, b). Additionally, in the skin of NRF2-deficient psoriasis-like mice, the STAT3 and NF-κB pathways were activated, and K16 expression increased (Fig. 8c, d). NAD(P)H dehydrogenase (NQO1), an NRF2-targeted enzyme possessing broad cytoprotective functions [30], was upregulated in the skin tissues of IMQ-induced psoriatic mice treated with SFN, whereas NRF2-deficient mice exhibited minimal expression (Fig. 8c, d). We utilized H&E staining and immunohistochemical techniques to observe the proliferation of mouse skin keratinocytes. We examined the epidermal thickness and detected the expression of K16, K17, and Ki67 to assess the proliferation of mouse skin keratinocytes, as in previous mouse experiments. Consistent with our expectations, NRF2 depletion promoted the cell cycle and proliferation of keratinocytes. Furthermore, SFN treatment did not yield significant differences (Fig. 8e, f). Considering that psoriasis is a Th17-related inflammatory disease, we assessed the expression of *Il17a* and *Il23a* in mouse skin tissues (Fig. 8g). In wild-type mice, SFN treatment led to a decrease in the expression of *Il17a* and *Il23a* in psoriatic mouse skin compared to the IMQ group. However, SFN did not achieve the same effect in NRF2-deficient mice. Moreover, in comparison to wild-type psoriatic mice, NRF2-deficient psoriatic mice exhibited an increase in the expression of *Il17a* and *Il23a* in skin tissues. These findings suggest that the therapeutic effect of SFN on IMQ-induced mice is primarily dependent on NRF2 activation, and that NRF2 deletion exacerbates psoriasis-like skin lesions (Fig. 9).

## Discussion

Bioactive compounds found in nature have great potential for treating diseases and may be safer in than synthetic agents [31, 32]. SFN is a natural isothiocyanate compound derived from plants, mainly from cruciferous vegetables such as cabbage, broccoli, cauliflower, and hugger kale, with a promising biosafety profile [33]. Previous studies have shown that SFN has antioxidant, antineoplastic, antiangiogenic, anti-inflammatory, and antibacterial properties [13, 34]. However, its function in psoriasis is poorly understood. Therefore, our current study aimed to investigate its therapeutic potential in psoriasis. Our study confirmed that SFN can be effective against cutaneous inflammation in an IMQ-induced psoriatic mouse model, consistent with previous findings of its therapeutic effects in chronic inflammatory diseases such as inflammatory bowel disease, respiratory diseases, and rheumatoid arthritis [35].

The current study found that SFN reduces psoriatic inflammation most likely due to its antioxidant properties. Several studies have found that oxidative stress is a significant contributor to psoriasis pathogenesis. Specifically, an imbalance between oxidants and antioxidants, increased ROS production, a reduction in antioxidant enzymes function contribute to the pathogenesis and development of psoriasis [3]. Oxidative stress biomarkers can be used to assess psoriasis severity, predict treatment outcome, and forecast comorbidities. Psoriasis symptoms are exacerbated by a variety of external pro-oxidative stress factors, including narcotics, smoking, drinking alcohol, stress, and bacterial infections [36, 37]. In our study, we demonstrated that SFN significantly alleviated psoriasis-associated oxidative stress injury, indicating its antioxidant properties.

To explore the molecular mechanism of SFN-mediated antioxidative protection, network pharmacology was used to predict the therapeutic targets of SFN in psoriasis. There were four enriched pathways identified in the pathway enrichment analysis, and the top-ranked pathway was KEAP1-NFE2L2. The KEAP1/NRF2 pathway demonstrates antioxidative and anti-inflammatory properties and plays a crucial role in maintaining skin homeostasis. Research suggests that NRF2 may play a role in promoting skin epidermal barrier formation and enhancing the differentiation of keratinocytes [38, 39]. The NRF2 activator, dimethyl fumarate, is clinically used to treat moderate to severe psoriasis [40], and various derivatives of it hold potential for treating psoriasis [41]. However, excessive activation of NRF2 could have detrimental effects. Therefore, further investigation into the role of NRF2 in psoriasis and the exploration of safe and effective NRF2 activators for psoriasis treatment are warranted. Our study revealed that NRF2 is highly expressed in healthy skin, which aligns with findings reported in public databases [42]. NRF2 activation emerges as a promising therapeutic approach for a range of skin disorders. Notably, we detected a marked reduction in NRF2 expression in the lesioned skin of both clinical biopsy samples and the IMQ-induced psoriatic mouse model. SFN treatment significantly restored the expression of NRF2 and alleviated the psoriatic pathological phenotype by blocking oxidative stress-induced skin tissue damage and cutaneous inflammation. The protective effect of SFN appears to be NRF2-dependent, as NRF2 deficiency exacerbated psoriasis-like symptoms, as well as the inflammatory response and abnormal proliferation of keratinocytes in the mice, and SFN had little therapeutic effect in the IMQ-modeled knockout mice. This finding supports our hypothesis that NRF2 plays a protective role in the development of psoriasis and that SFN acts via NRF2-related pathways. In in vitro experiments, NRF2 expression was reduced in IL-22-stimulated HaCaT cells, while the addition of SFN partially restored NRF2 expression. We also noticed that SFN treatment reduced IL-22-induced ROS production in HaCaT cells.

Another important mechanism for SFN-mediated immune protection against psoriatic inflammation is its function in the regulation of inflammatory pathway activation. JAK2/STAT3 has recently been recognized as a key inflammatory pathway in the development and pathogenesis of psoriasis. STAT3 not only involved in promoting Th17 cell differentiation but also directly activates IL-17 [43]. TNF-α/NF-κB pathway is another important inflammatory pathway in the development of psoriasis. Inhibitors of this pathway, such as etanercept, infliximab, and adalimumab, have proven effective in clinical practice [44]. In our study, we treated psoriasis-induced mice and IL-22- or TNF-α-stimulated HaCaT cells with SFN and subsequently measured the activation status of the STAT3 and NF-κB pathways. Our results demonstrated a significant reduction in inflammatory pathway activation in both the IMQ-induced psoriasis mouse model and the in vitro cell model. Another characteristic of psoriasis is the excessive proliferation and aberrant terminal differentiation of keratinocytes. The upregulation of K16 and K17 in keratinocytes plays a crucial role in modulating the proliferation, migration, and inflammatory response of keratinized cells [45, 46]. As a result, K16 and K17 are generally considered as biomarkers and potential therapeutic targets for psoriasis. We assessed keratin expression and found that the elevated expression of K16 and K17 diminished in both in vivo and in vitro experiments following SFN treatment. In line with these findings, we discovered that SFN could inhibit the expression of IL-6 and IL-1β, both of which are regulated by NRF2 [47, 48]. However, our cellular and mouse modeling approaches closely resemble the acute inflammatory phase of psoriasis development. Further research is needed to investigate the therapeutic effects of sulforaphane and NRF2 in chronic psoriasis models and in patients with psoriasis.

## Conclusion

Our study reveals that reduced expression of NRF2 contributes to oxidative stress injury, which plays a role in the pathogenesis of psoriasis. Treatment with SFN activates the NRF2 signaling pathway, mitigating oxidative stress injury and attenuating the inflammatory response associated with psoriasis. These findings suggest that SFN holds promise as an effective therapeutic agent for alleviating cutaneous inflammation in psoriasis.

### Supplementary information

information of Figure S1

Original Data File

reproducibility checklist

## Data Availability

The data used to support the findings of this study are included within the article.
