# Supplementary material for: Sulforaphane alleviates psoriasis by enhancing antioxidant defense through KEAP1-NRF2 Pathway activation and attenuating inflammatory signaling
Source: Cell Death Dis. 2023 Nov 25;14(11):768. doi: 10.1038/s41419-023-06234-9 (PMC10676357; doi:10.1038/s41419-023-06234-9)
Supplement: Supplementary file 1 — information of Figure S1 [file 41419_2023_6234_MOESM1_ESM.docx]

**Supplementary Materials for**

**Sulforaphane alleviates psoriasis by enhancing antioxidant defense through KEAP1-NRF2 Pathway activation and attenuating inflammatory signaling**

**MA et al. 2023**


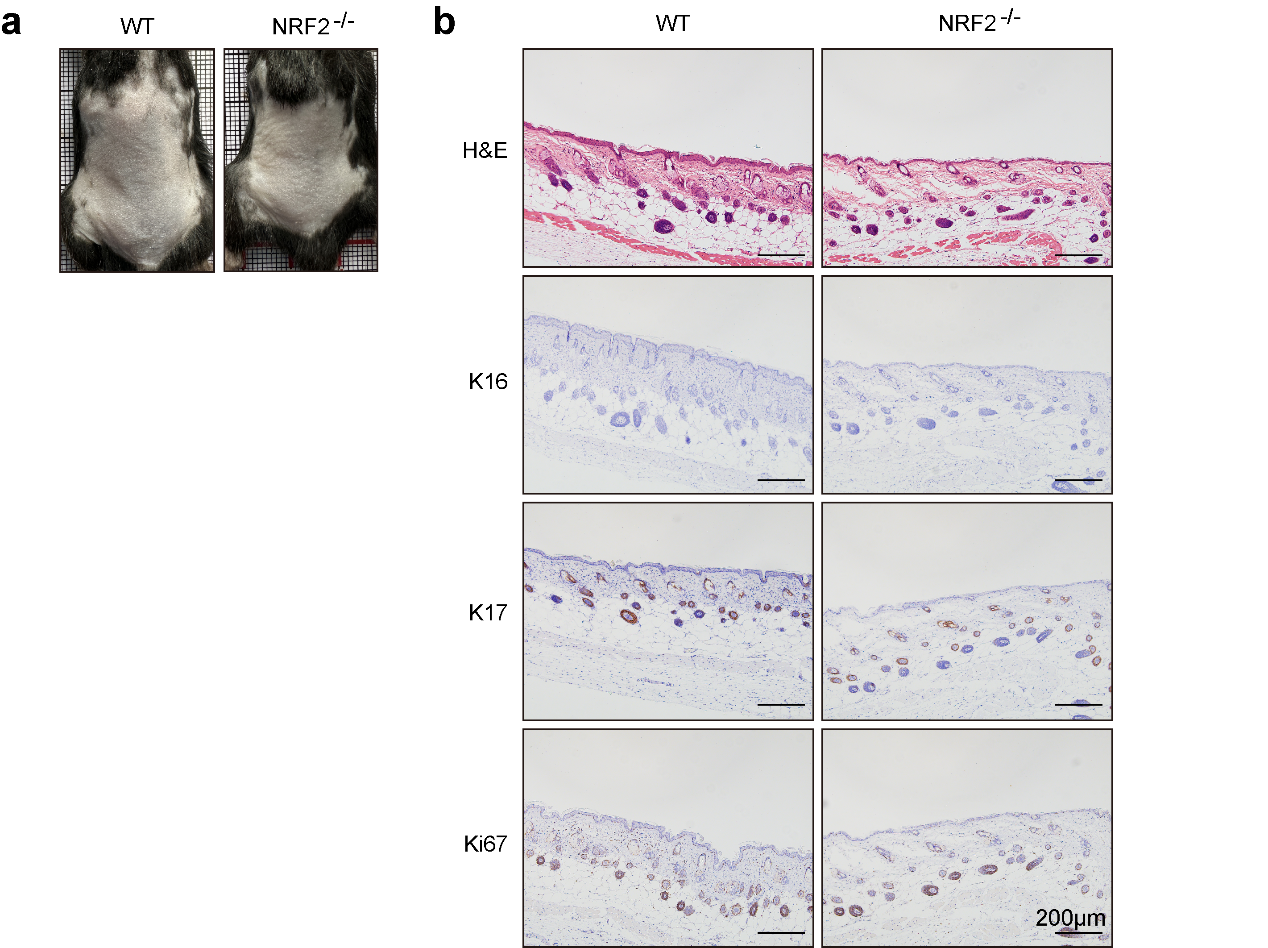


**Figure S1. The skin of NRF2-deficient mice showed no significant difference compared to the control group mice in the absence of modeling.** (**A**) Wild-type mice and NRF2^−/−^ mice had their dorsal fur removed and were then treated topically with Vaseline for 7 days. Images were taken on day 8. (**B**) H&E staining and immunohistochemical staining for Ki67, K17, and K16 were conducted on skin sections of mice at a magnification of ×100.
