# Supplementary material for: Sulforaphane alleviates psoriasis by enhancing antioxidant defense through KEAP1-NRF2 Pathway activation and attenuating inflammatory signaling
Source: Cell Death Dis. 2023 Nov 25;14(11):768. doi: 10.1038/s41419-023-06234-9 (PMC10676357; doi:10.1038/s41419-023-06234-9)

Figure 2b
p-STAT3

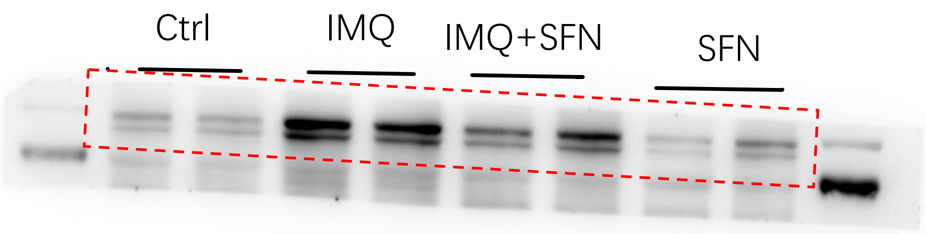

Figure 2b
STAT3

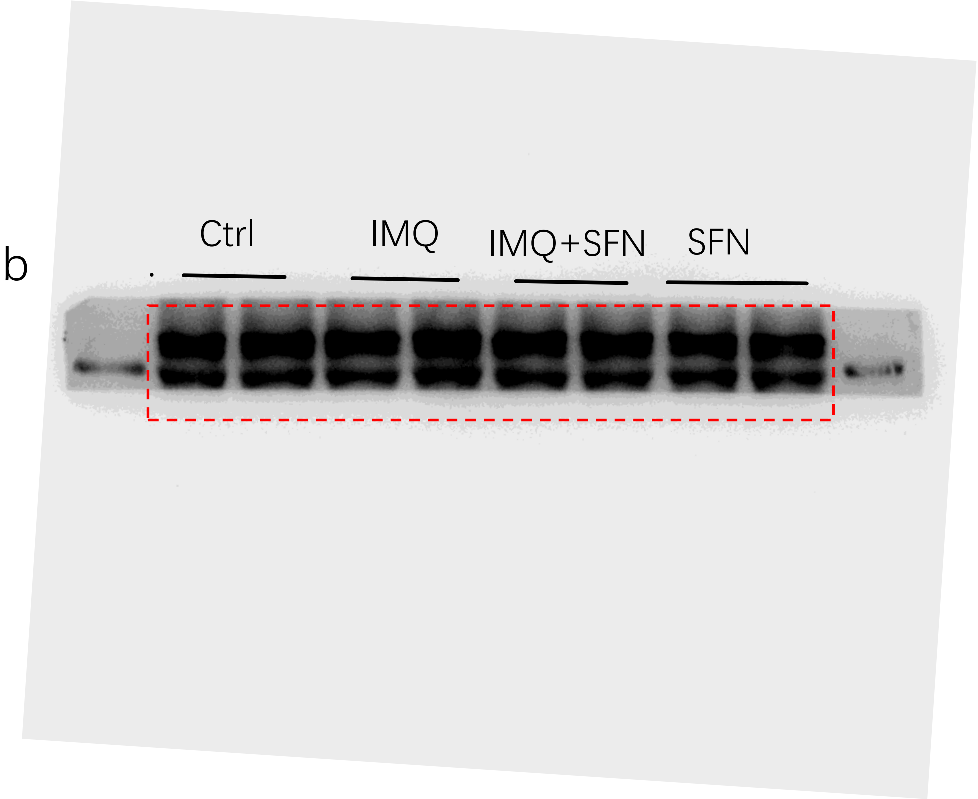

Figure 2b
p-IκB

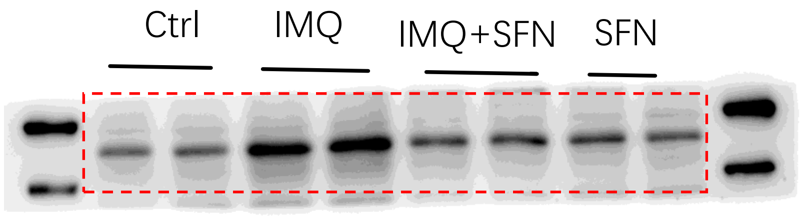

Figure 2b
IκB

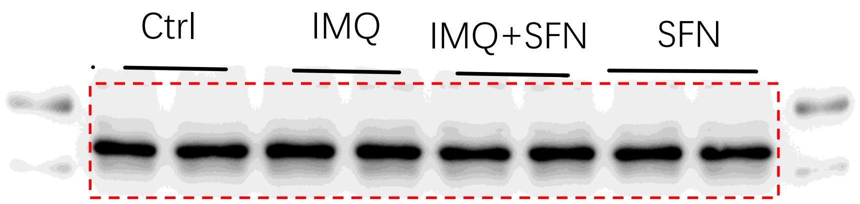

Figure 2b
K16

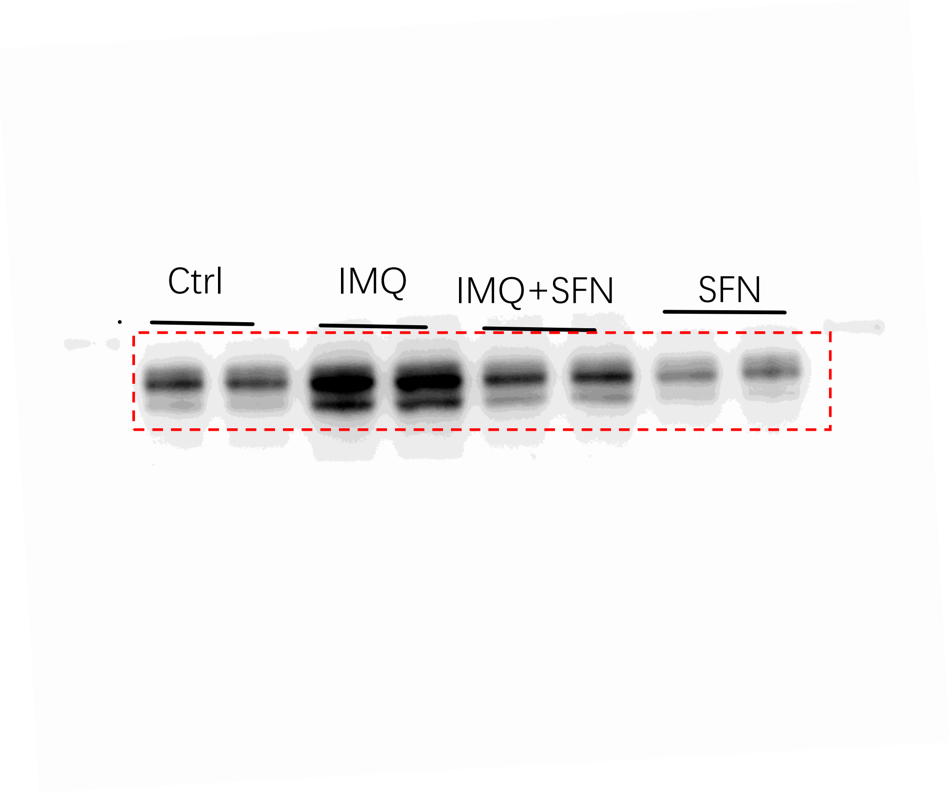

Figure 2b
K17

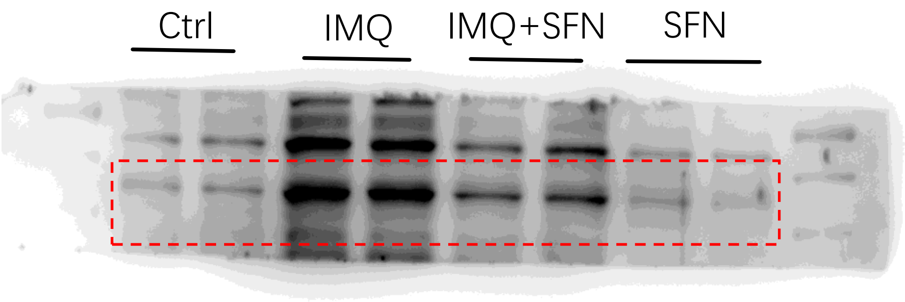

Figure 2b
GAPDH

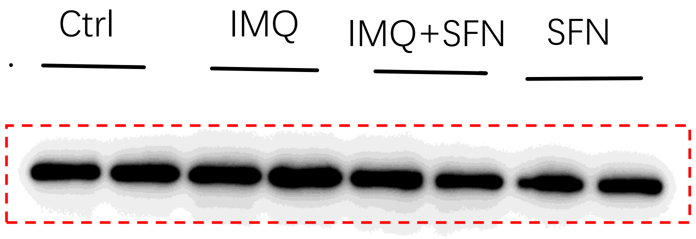

Figure 3a
p-STAT3

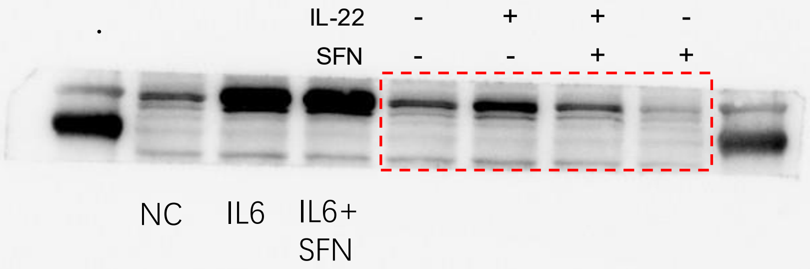

Figure 3a
STAT3

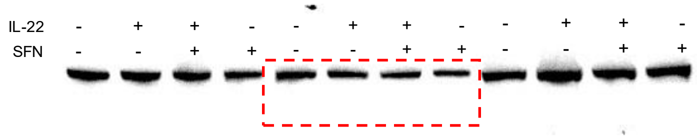

Figure 3a
K16

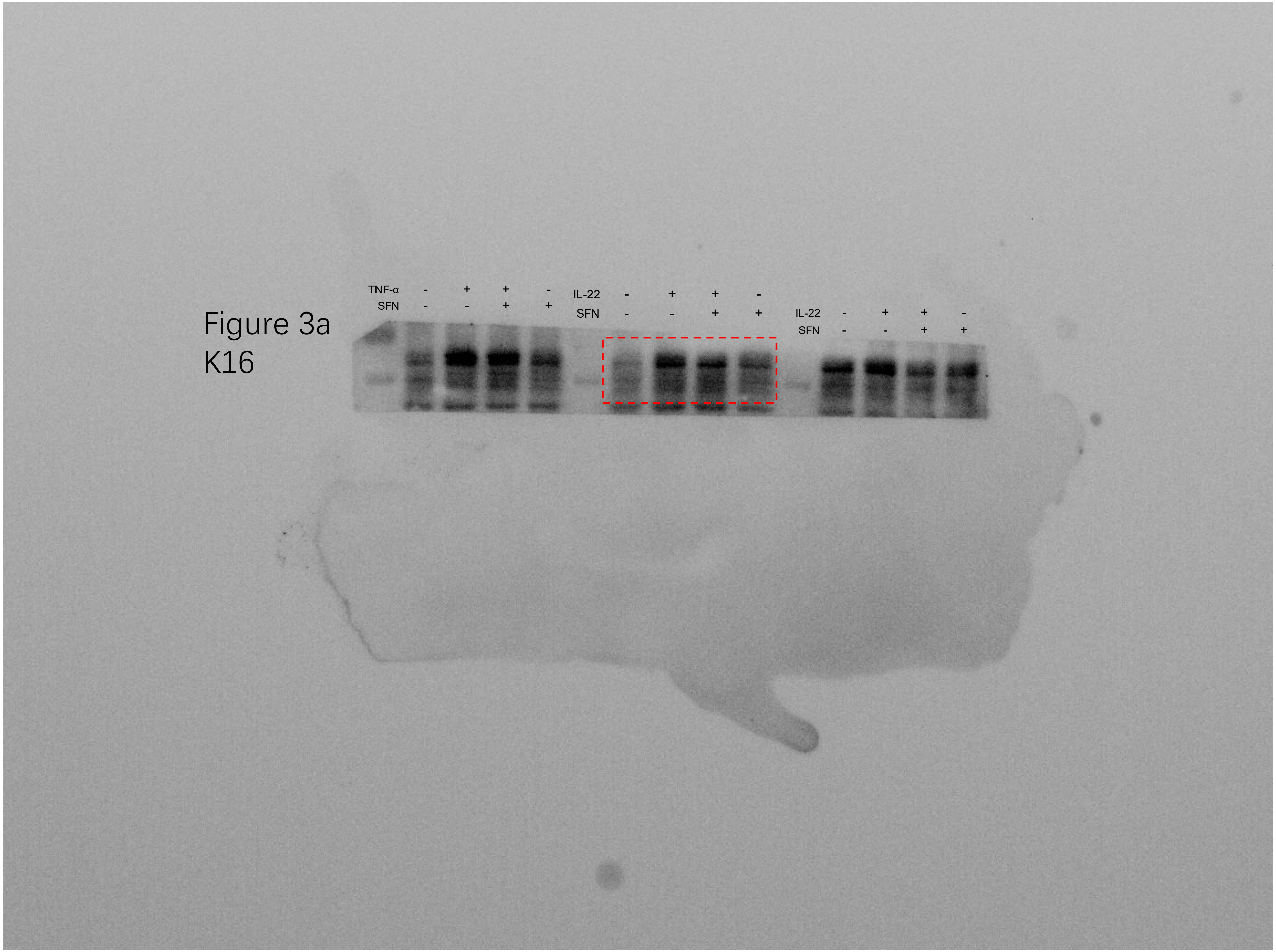

Figure 3a
K17

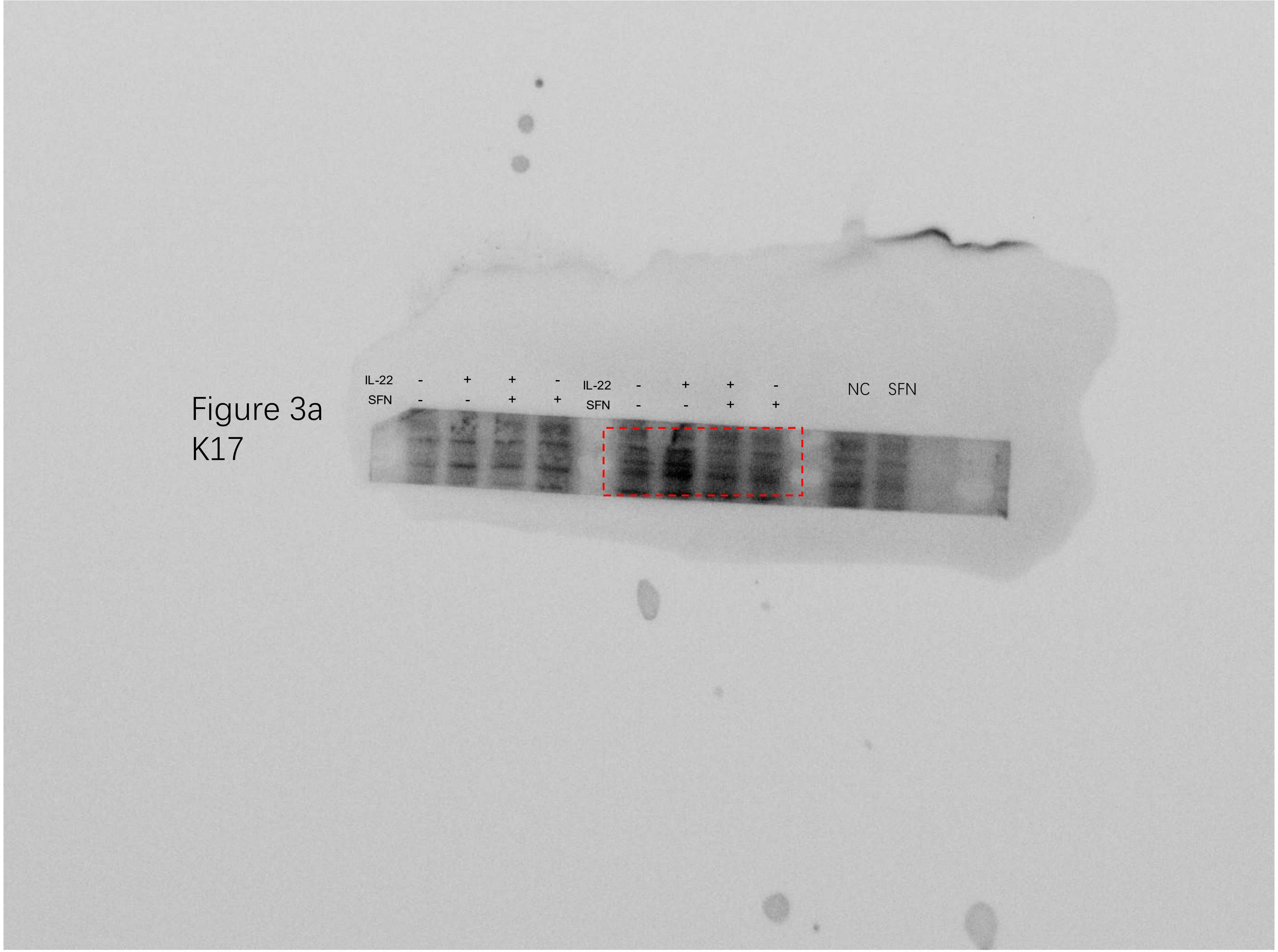

Figure 3a
GAPDH

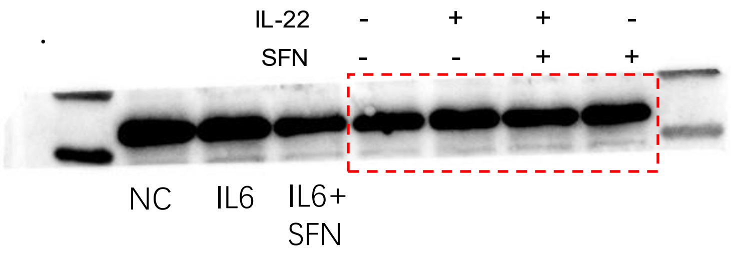

Figure 3b
p-IκB

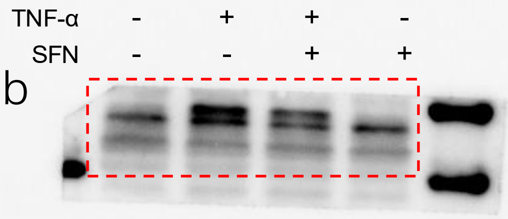

Figure 3b
IκB

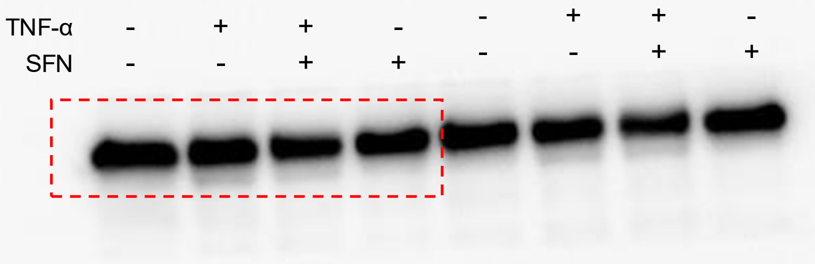

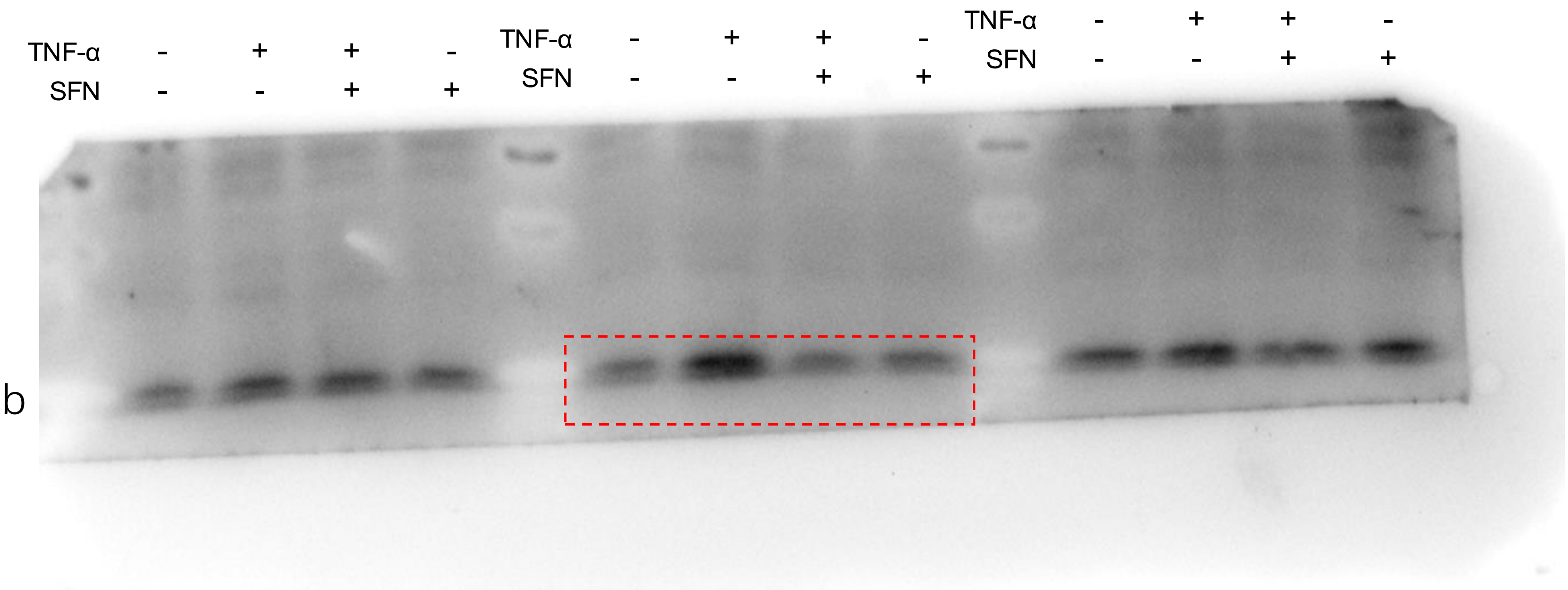

Figure 3b
IL-1β

Figure 3b
K16

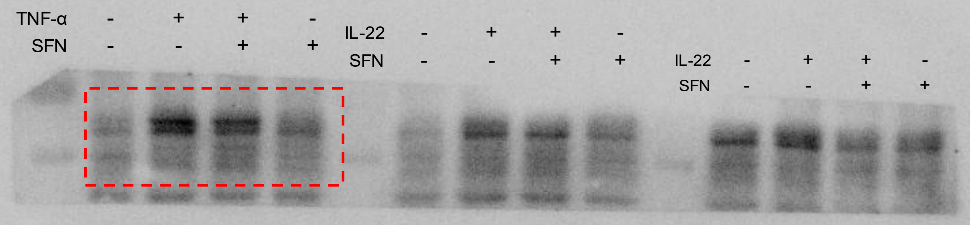

Figure 3b
K17

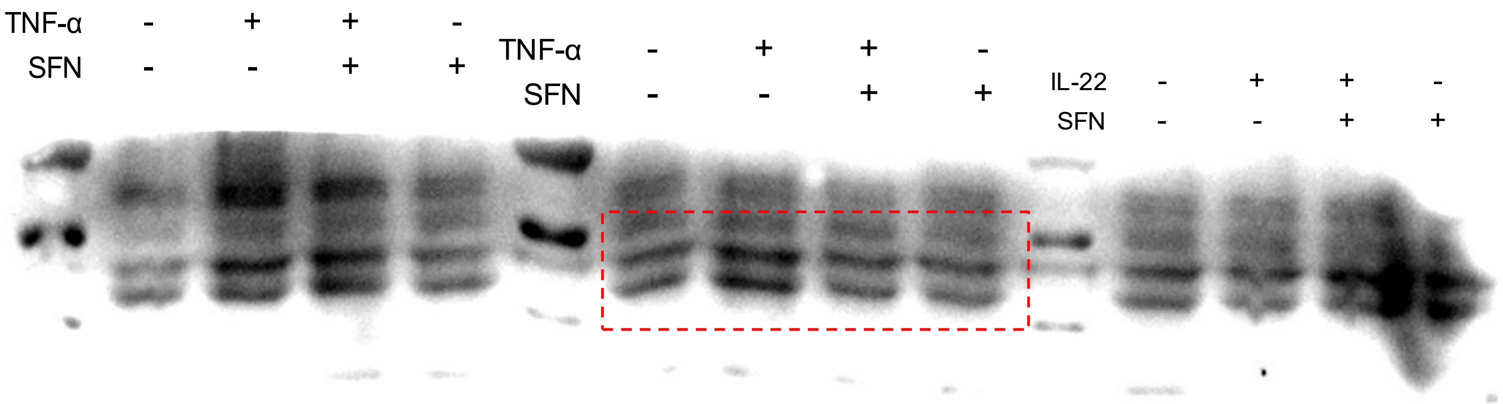

Figure 3b
GAPDH

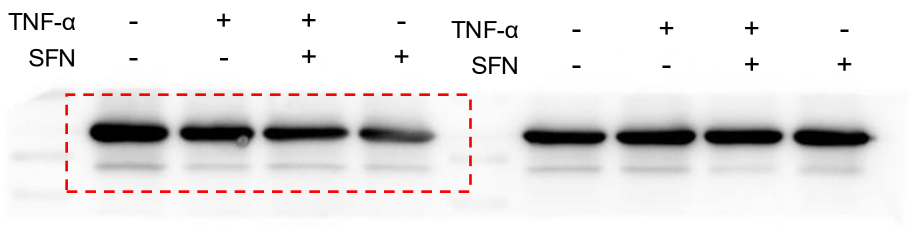

Figure 6a
NRF2

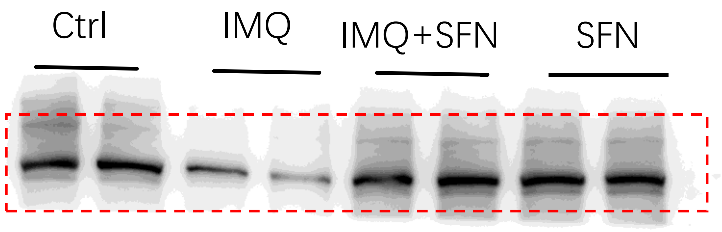

Figure 6a
p-NRF2

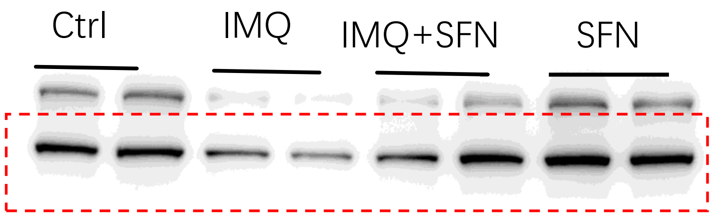

Figure 6a
GAPDH

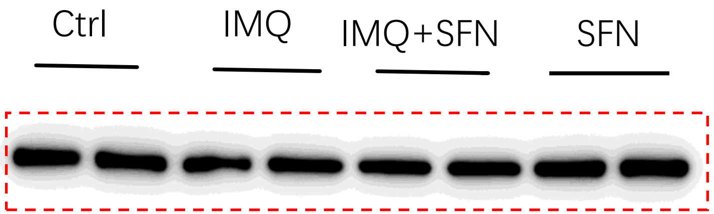

Figure 7a
NRF2

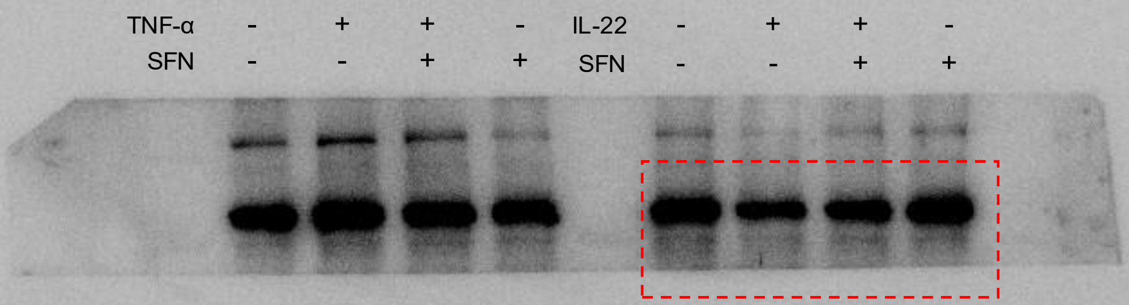

Figure 7a,7b
p-NRF2

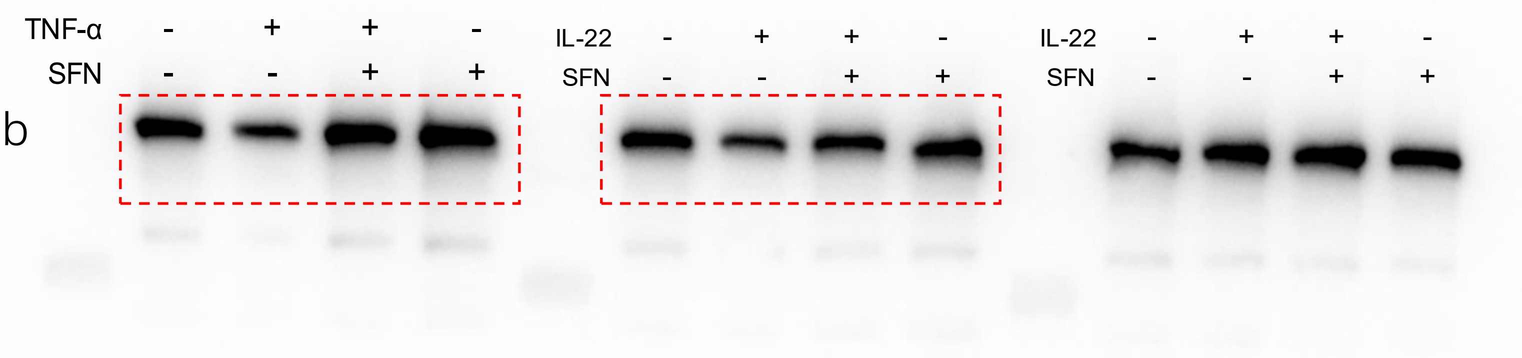

Figure 7b
NRF2

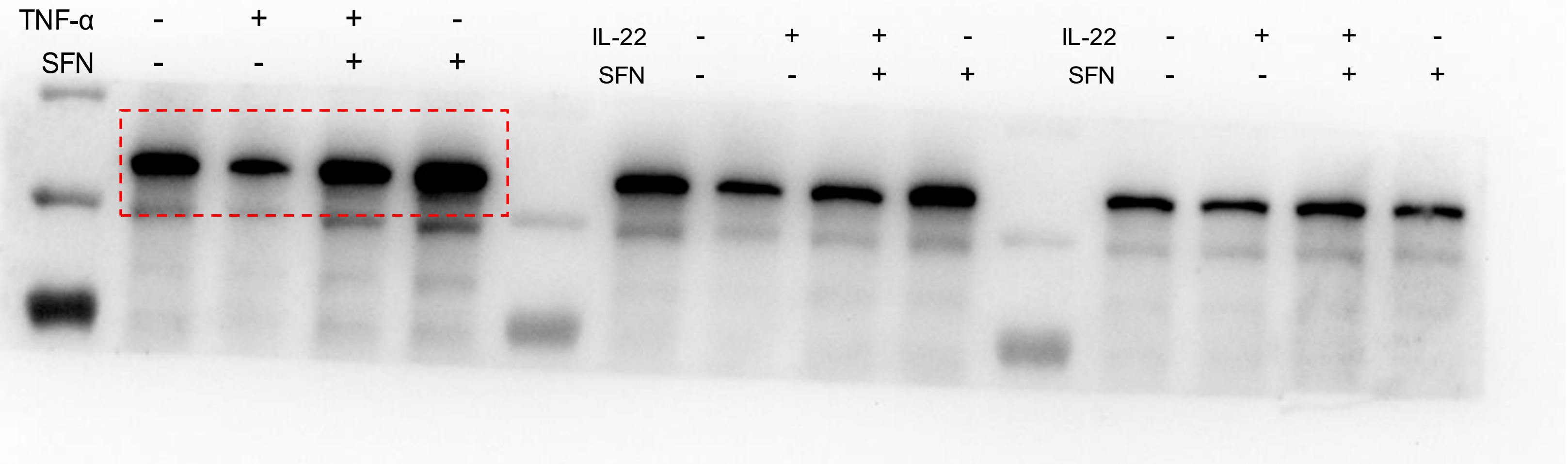

Figure 7a
GAPDH

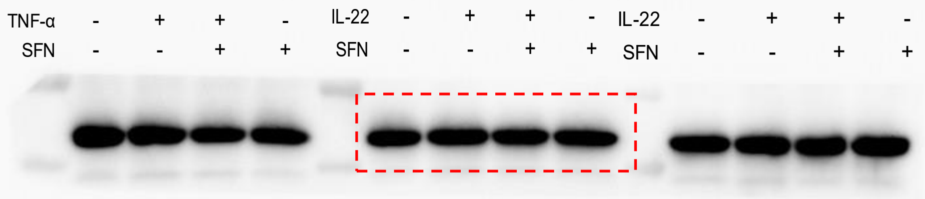

Figure 7b
GAPDH

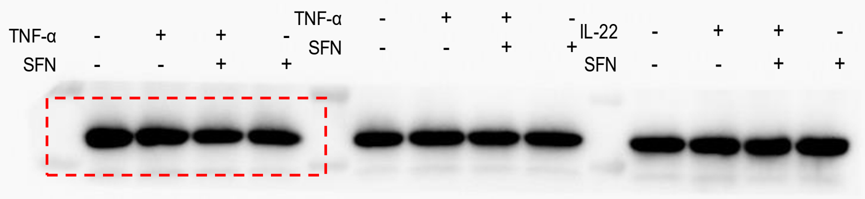

Figure 7e
NRF2

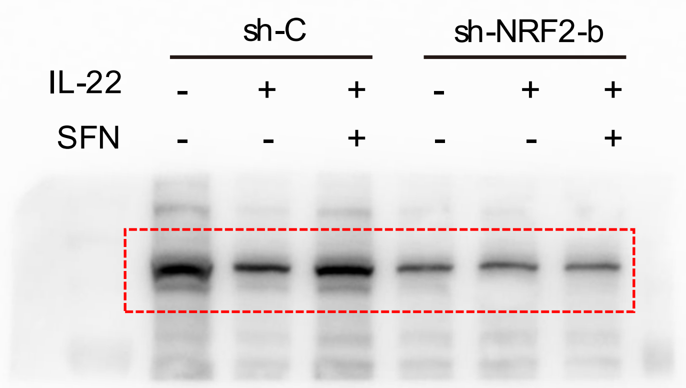

Figure 7e
NQO1

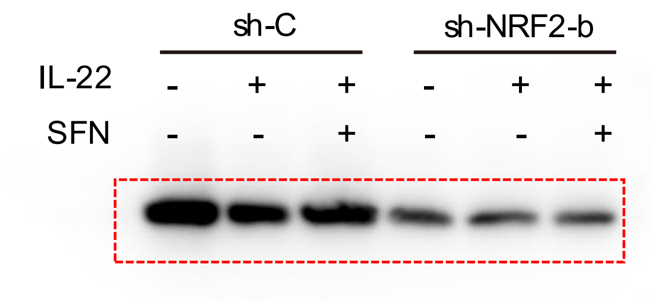

Figure 7e
p-STAT3

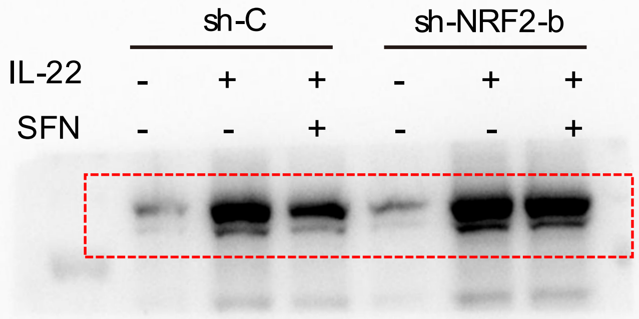

Figure 7e
STAT3

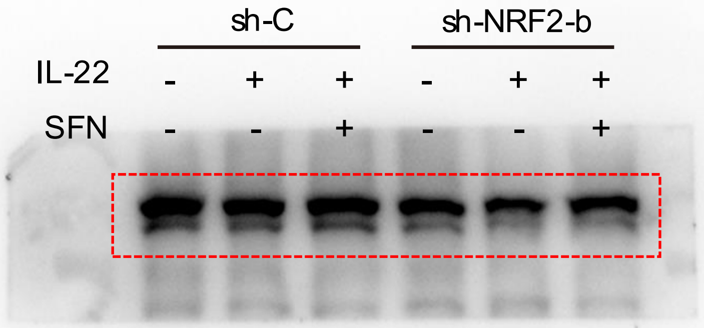

Figure 7e
K16

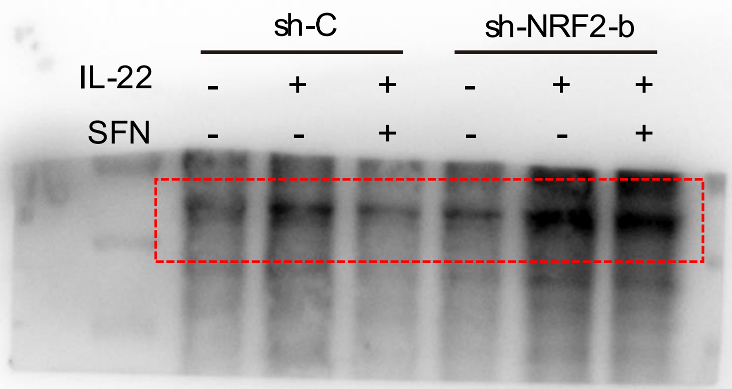

Figure 7e
K17

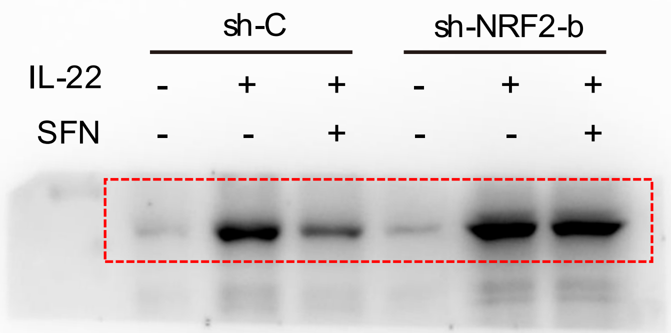

Figure 7e
GAPDH

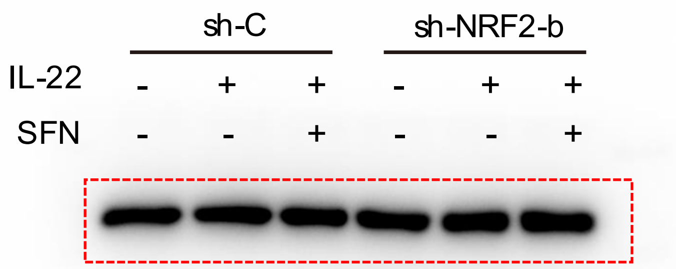

Figure 7f
NRF2

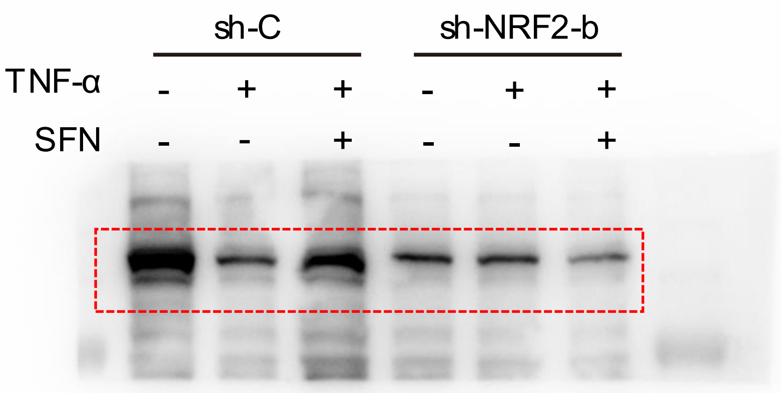

Figure 7f
NQO1

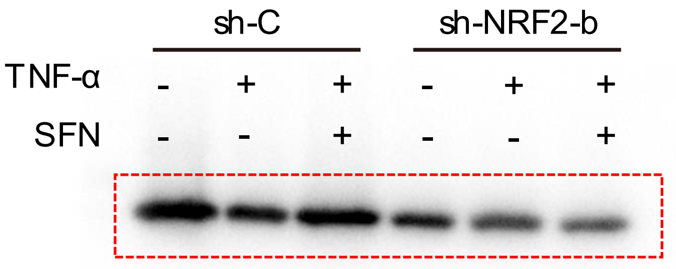

Figure 7f
p-IκB

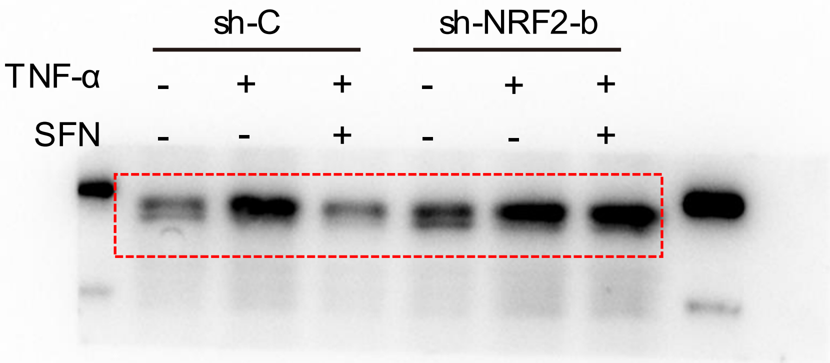

Figure 7f
IκB

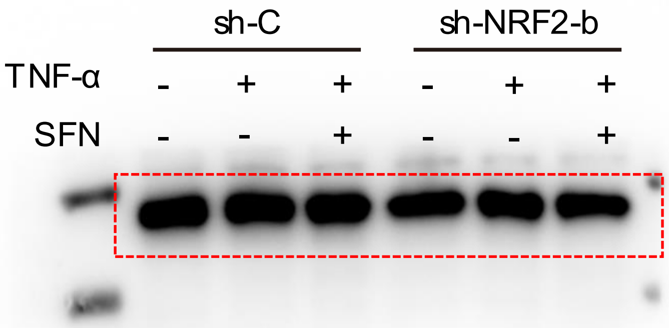

Figure 7f
K16

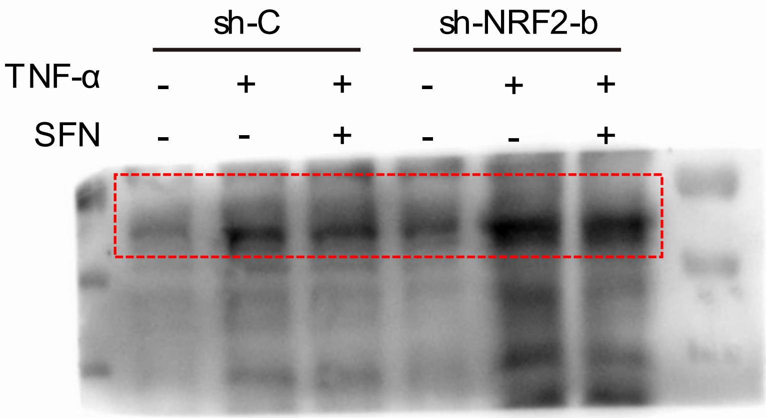

Figure 7f
K17

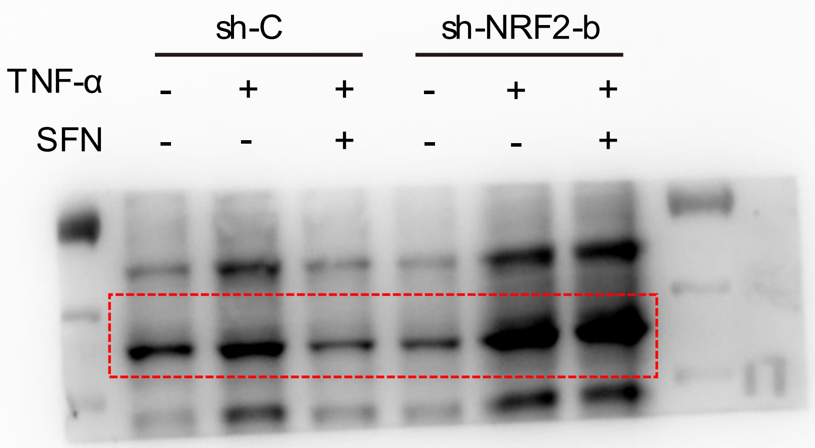

Figure 7f
K16

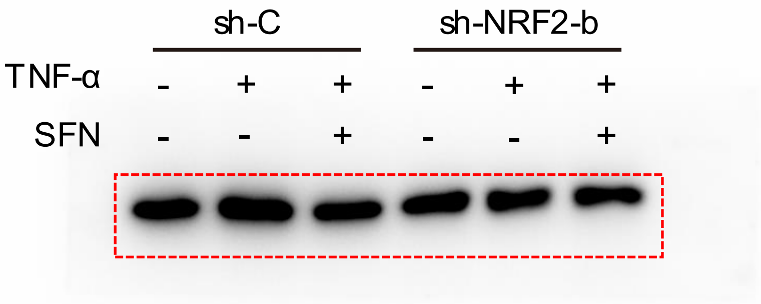

Figure 8c
NRF2

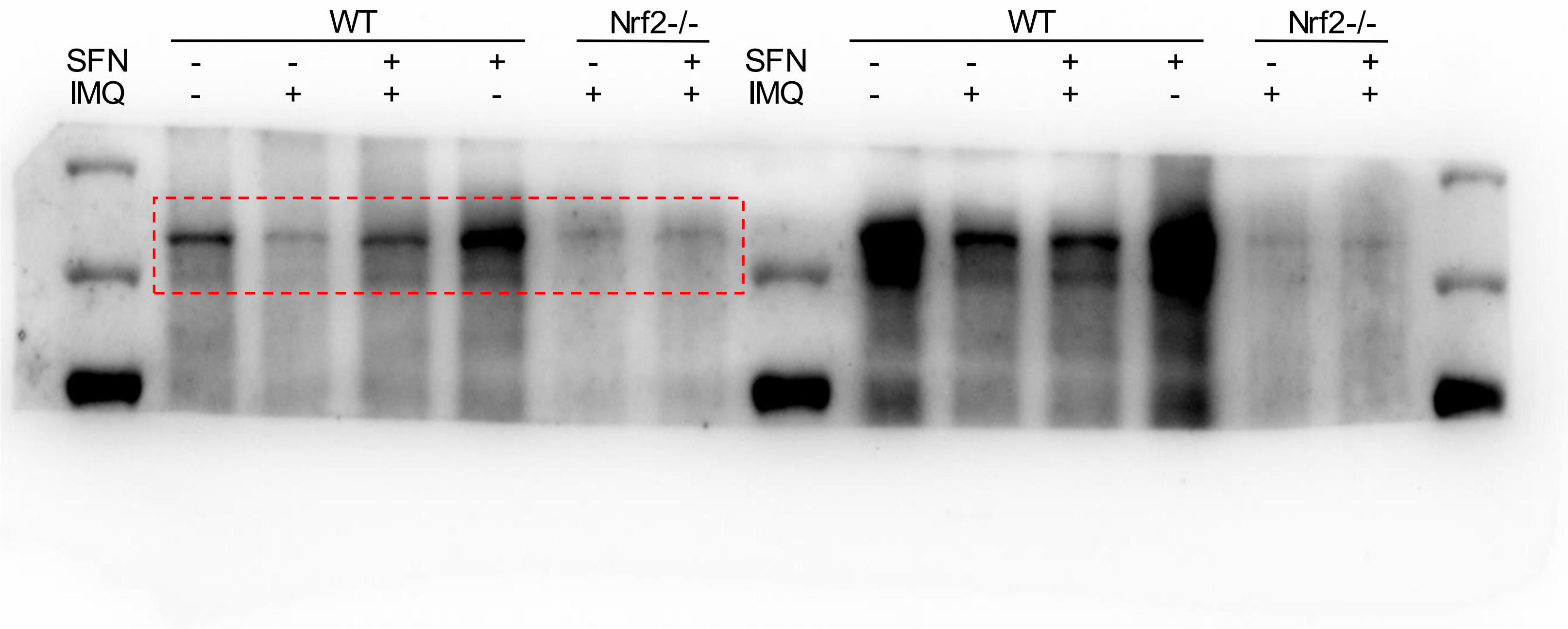

Figure 8c
p-STAT3

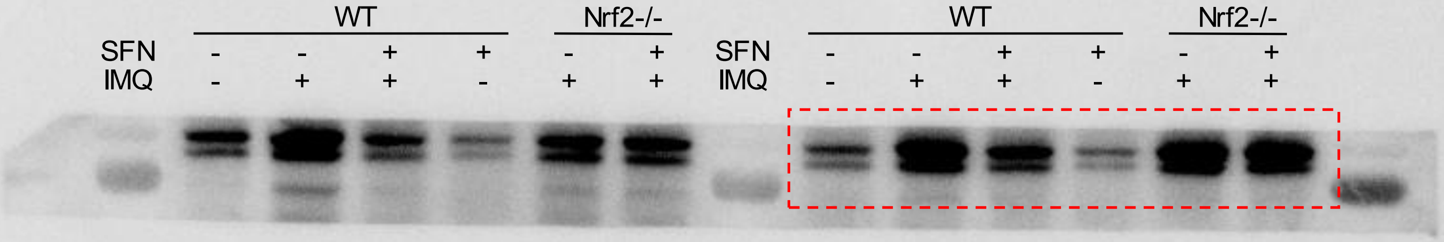

Figure 8c
STAT3

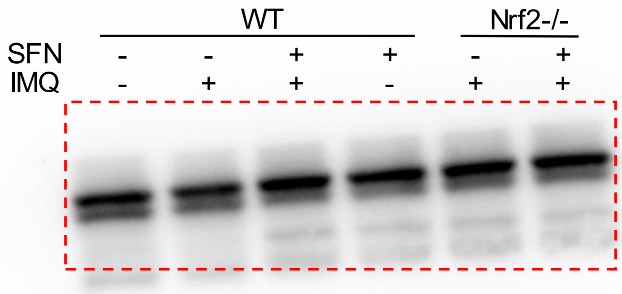

Figure 8c
p-P65

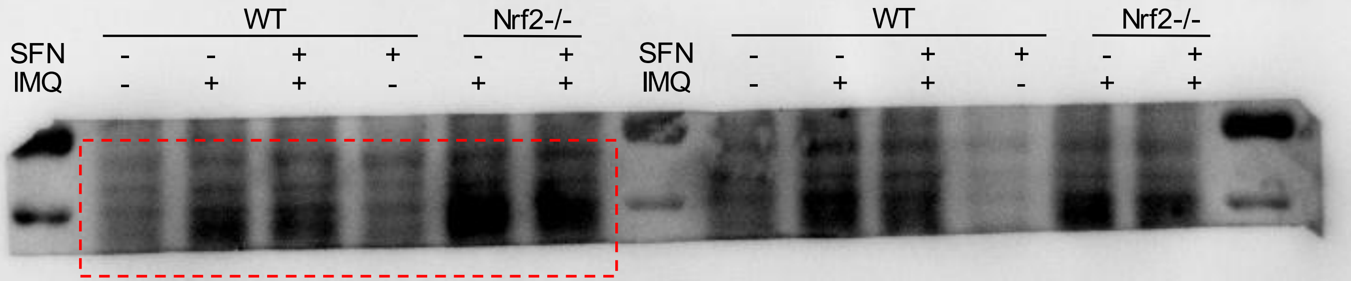

Figure 8c
P65

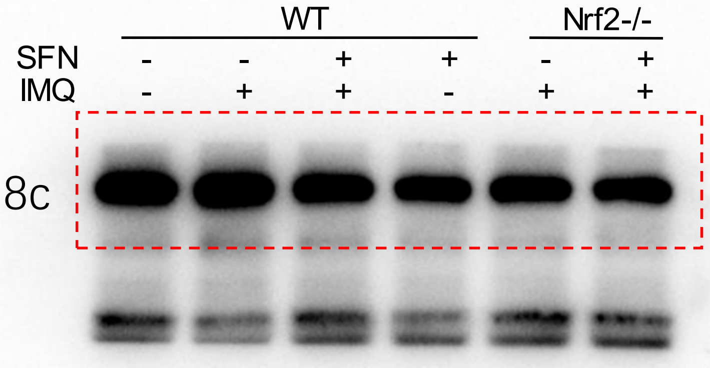

Figure 8c
p-IκB

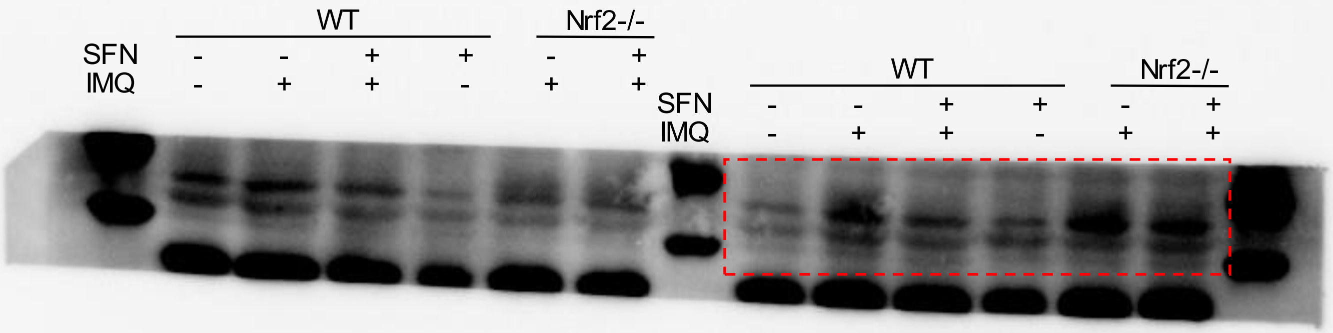

Figure 8c
IκB

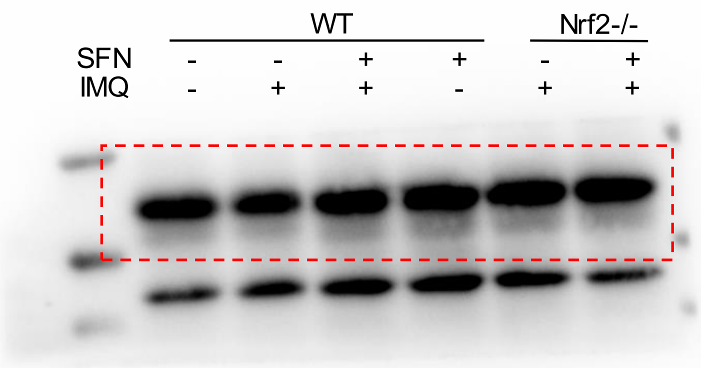

Figure 8c
K16

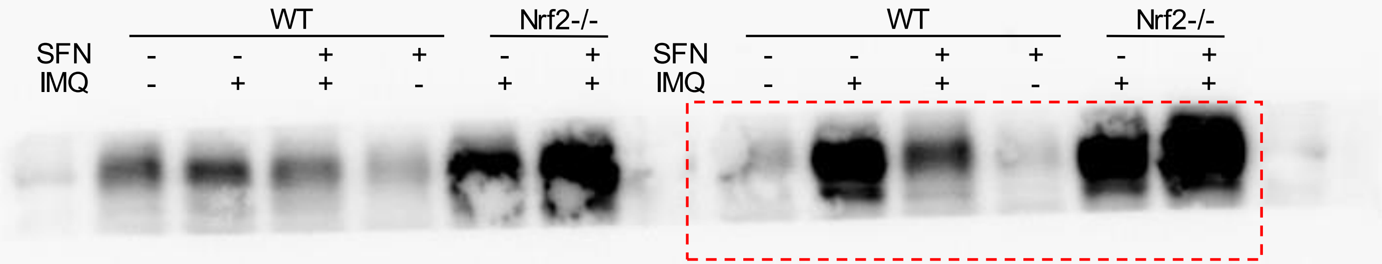

Figure 8c
NQO1

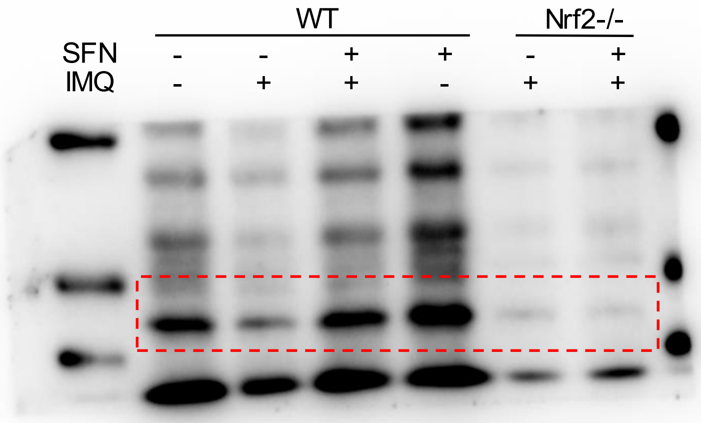

Figure 8c
GAPDH

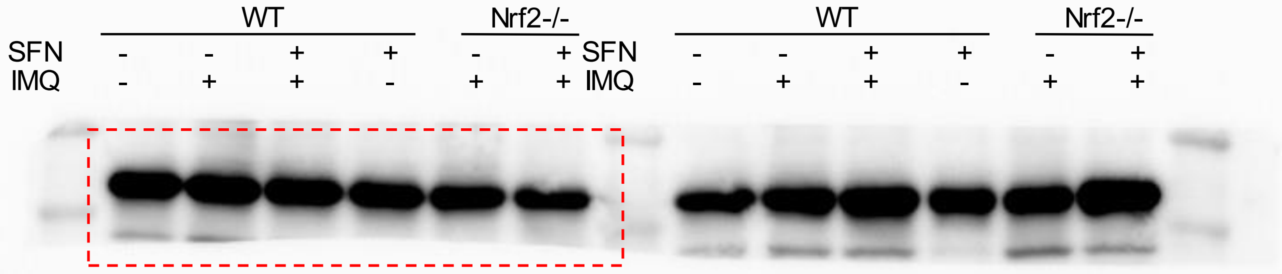

Supplement: Supplementary file 2 — Original Data File [file 41419_2023_6234_MOESM2_ESM.pdf]
